# Human Endogenous Retrovirus Protein Activates Innate Immunity and Promotes Experimental Allergic Encephalomyelitis in Mice

**DOI:** 10.1371/journal.pone.0080128

**Published:** 2013-12-06

**Authors:** Hervé Perron, Hei-Lanne Dougier-Reynaud, Christina Lomparski, Iuliana Popa, Reza Firouzi, Jean-Baptiste Bertrand, Suzana Marusic, Jacques Portoukalian, Evelyne Jouvin-Marche, Christian L. Villiers, Jean-Louis Touraine, Patrice N. Marche

**Affiliations:** 1 Geneuro, Plan-les-Ouates, Geneva, Switzerland; 2 Geneuro-Innovation, Lyon, France; 3 Université Lyon-1, Lyon, France; 4 Université Joseph Fourier, Grenoble, France; 5 Institut Albert Bonniot, UMR_S823, Université Joseph Fourier, Grenoble, France; 6 ImmunAlp, Gières, France; 7 UMR CNRS 8612, University of Paris XI, Chatenay Malabry, France; 8 Hook laboratories, Lawrence, Massachusetts, United States of America; 9 Hospices Civils de Lyon, Lyon, France; Institute Biomedical Research August Pi Sunyer (IDIBAPS) - Hospital Clinic of Barcelona, Spain

## Abstract

Multiple sclerosis (MS) is a complex multifactorial disease of the central nervous system (CNS) for which animal models have mainly addressed downstream immunopathology but not potential inducers of autoimmunity. In the absence of a pathogen known to cause neuroinflammation in MS, Mycobacterial lysate is commonly used in the form of complete Freund's adjuvant to induce autoimmunity to myelin proteins in Experimental Allergic Encephalomyelitis (EAE), an animal model for MS. The present study demonstrates that a protein from the human endogenous retrovirus HERV-W family (MSRV-Env) can be used instead of mycobacterial lysate to induce autoimmunity and EAE in mice injected with MOG, with typical anti-myelin response and CNS lesions normally seen in this model. MSRV-Env was shown to induce proinflammatory response in human macrophage cells through TLR4 activation pathway. The present results demonstrate a similar activation of murine dendritic cells and show the ability of MSRV-Env to trigger EAE in mice. In previous studies, MSRV-Env protein was reproducibly detected in MS brain lesions within microglia and perivascular macrophages. The present results are therefore likely to provide a model for MS, in which the upstream adjuvant triggering neuroinflammation is the one detected in MS active lesions. This model now allows pre-clinical studies with therapeutic agents targeting this endogenous retroviral protein in MS.

## Introduction

Multiple sclerosis is a complex multifactorial disease of the central nervous system, which involves environmental factors and genetic susceptibility as upstream factors [1]. As these aetiological factors have not yet been clearly defined, MS studies have predominantly been focused on the resulting pathogenic process involving neuroinflammation, demyelination, axonal damage and defects in myelin repair [2], [3], [4]. Animal models for MS have also been dedicated to the study of downstream effectors of anti-myelin autoimmunity and neuroinflammation thus developing experimental allergic encephalomyelitis (EAE) as the unique model for pre-clinical therapeutic assessment for MS [5], [6]. As no “upstream” pathogen responsible for the induction of demyelinating neuroinflammatory lesions of MS had been identified, a “heterologous” pathogen extract, i.e. mycobacterial lysate with peculiar immunoadjuvant properties capable of promoting auto-immunity against artificially presented myelin antigens, has been used in the form of mineral oil suspension designated as ‘complete Freund's adjuvant’ -CFA- [7], [8]. This has permitted the elucidation of many immunological functions that could lead to immune-mediated demyelination and, consequently, has delineated the therapeutic scope for MS within the boundaries of immunological effectors [9], [10], [11].

Following the discovery of retroviral RNA sequences from Multiple Sclerosis associated RetroViral element (MSRV) in virion particles associated with reverse-trancriptase activity from MS cell cultures [12], [13], a previously unknown family of human endogenous retroviruses (HERV) was identified (HERV-W) [14]. Families of “endogenous” retroviruses refer to what is now known to represent 8% of the human genome and to result from ancestral retroviral infections that have integrated proviral genomes in chromosomes of germ-line cells [15]. These retroviral sequences are part of the human genome but have an uneven distribution in the population [16], [17], [18]. Although the great majority of them are inactivated by genetic alterations and the few potentially active elements with open reading frames (orf) are usually epigenetically silenced, non-physiological expression of retroviral genes and particles have been reported in several human diseases [19].

Concerning the HERV-W family and its virion-associated MSRV element, an association with multiple sclerosis has been repeatedly and independently demonstrated over the past two decades [12], [18], [20], [21], [22], [23], [24], [25], [26], [27].

Moreover, as the expression of HERV-W elements in MS could have been a consequence of the disease process without pathogenic effect, studies have addressed the potential pathogenicity of MSRV particles and of corresponding proteins on immune and glial cells. Those studies have shown a pathogenic effect on glial cells [28] and the resulting cellular effects impairing remyelination have only been recently elucidated [29]. Most importantly, they have also shown a potent immunopathogenic effect of MSRV virions caused by the envelope protein (MSRV-Env) on T-lymphocytes [30] with a predominant upstream activation of innate immunity specifically mediated by the TLR4 receptor and its CD14 co-receptor [31]. These results have consequently made it obvious that this HERV-W expression producing MSRV particles and/or proteins would not be neutral to surrounding cells and could play a role in the MS pathogenic process.

Data from successive and independent studies showed the presence of HERV-W/MSRV-Env protein (using detection antibodies specific for highly conserved HERV-W Env proteins) on macrophages or microglial cells in active plaques of all MS brains studied to date [20], [25], [26], [32]. This has led to the hypothesis that MSRV-Env expression may be pivotal in the initiation of pathogenic process leading to MS lesions.

In order to further explore this possibility, we have evaluated whether MSRV-Env protein could trigger EAE development in mice if used instead of mycobacterial lysate of Complete Freund's adjuvant. The protein was expressed either from the full-length open reading frame (orf) of an MSRV-env clone or from a truncated portion of the protein comprising its surface domain (SU) containing the TLR4-stimulating region. The present study demonstrates that MSRV-Env protein from the HERV-W family is efficient in inducing EAE in C57/BL6 mice when administered in emulsion together with myelin oligodendrocyte glycoprotein (MOG) peptide 35–55. MSRV-Env-induced disease developed with clinical and histopathological features indistinguishable from those of standard EAE. These results support an *in vivo* pathogenicity of this peculiar HERV-W protein and suggest that it may play a similar pathogenic role in MS.

## Materials and Methods

All animal work has been conducted according to relevant national and international guidelines. The protocol was approved by the Committee on the Ethics of Animal Experiments of Lyon-1 University (Permit Number: BH2009-09)and all efforts were made to minimize suffering.”

### Proteins and toxins

Recombinant Env protein was produced for Geneuro by Protein'Expert (Grenoble, France) from plasmid pV14 [30] encompassing the complete orf of HERV-W envelope protein (58 KDa, 542 aminoacids) cloned from MSRV virion RNA (Env; GenBank no. AF331500.1). Env-SU is a 33-kDa and 293 amino acids fractions of the full-length MSRV-Envelope protein (542 amino acids). The recombinant Env-SU protein was produced and purified by Protein'eXpert (Grenoble, France) as previously described (Rolland et al. 2006). The proteins were refolded and biologically active. As a negative control, a mock protein was synthesized and purified under the same conditions. Fifty-microgram aliquots per -milliliter were flash frozen into liquid nitrogen and then stored at −80°C. Quality and purity of the recombinant proteins were assessed by mass spectroscopy and western-blot. The presence of endotoxins in proteins preparations was tested by a *Limulus* -amebocyte -lysate (LAL) test performed by CleanCells (Bouffere, France), and all production batches were below the detection level of 5 UI/ml.

Details on env protein sequence, domains and productions are also provided in Figure S22 and QC reports in File S1.

Mouse mAb specific for HERV.W proteins were provided by GeNeuro SA (Geneva, Switzerland): GN-mAb_03 is targeted against Env-SU protein and GN-mAb12 against gag protein. Myelin Oligodendrocyte Glycoprotein (MOG) peptide MOG 35–55 was obtained from (Enzo Life Science AG, Lausanne, Switzerland) and *Bordetella pertussis* toxin from (Sigma-Aldrich, France).

### Mice

For Env-SU series, pathogen-free 6 week-old wild-type C57BL/6 mice were purchased from Janvier (France). TLR4 knockout (KO) mice and CD14 KO mice were obtained from Dr. Bernard Ryffel from the Centre National de la Recherche Scientifique/Centre de Distribution, Typage et Archivage Animal (CNRS, Orléans, France). For replication series with entire Env protein, as presented here, 10 weeks old C57BL/6 mice were purchased from Taconic Farms, USA.

### DC culture and stimulation

DC were generated from bone marrow (BM) as described (Berthier et al., 2000). Briefly, BM cells were isolated by flushing cells from the femurs. Erythrocyte and Gr1^+^ cells were removed by magnetic cell sorting. Negatively selected cells were resuspended at 5×10^5^ cells/ml in complete Iscove's modified Dulbecco's medium (IMDM) (Gibco, Invitrogen, Grand Island, NY, USA) supplemented with 1% of -GM-CSF-transfected J558 cell line supernatant, 40 ng/ml of mouse recombinant FLT-3L, and 5 ng/ml of mouse recombinant IL-6. Every 3 days, cells were resuspended at 5×10^5^ cells/ml in complete IMDM. By day 6, IL-6 was removed and FLT-3L was used at 20 ng/ml. BM-DC were plated in 24-well plates at a concentration of 1×10^6^/well in 1 ml of culture medium after stimulation with LPS and different amounts of Env-SU: cells were incubated at 37°C in 5% CO_2_ in humidified atmosphere for 24 h. For inhibition experiments with mAb, 1 µg of Env-SU and LPS was preincubated for 45 min at 4°C with 30 µg/ml of GN-mAb_03 anti Env mAb or of GN-mAb_12 anti gag mAb as control prior addition to DC culture.

### Cellular analysis

For flow cytometry analysis, cells were harvested, washed in PBS, 3% FCS, 0.16% sodium azide and immunostained for surface expression of distinct markers for 30 min at 4°C. The conjugated Ab used for staining were all obtained from BD Biosciences: IAB-PE, CD11c-APC. Data acquisition and analysis were performed with a FACS Aria flow cytometer and the software FACSDiva5 (BD Biosciences). A propidium iodide staining has been done in order to exclude dead cells.

### Cytokine production assays

Culture supernatants were collected and concentrations of the proinflammatory cytokines IL-6, IL-12p70, γIFN, and TNF-α were measured using ELISA kits (BD Pharmingen, eBioscience), according to manufacturer's instructions.

### EAE induction in mice

C57BL/6 female mice (6–8 weeks of age) were acclimated in the animal facility for two weeks and were immunized (day 0) subcutaneously at 2 sites of the neck and in the flanks (day 7 and day 14) with 200 µg of MOG 35–55 (NeoMPS, Strasbourg) per animal either 1) emulsified in complete Freund's adjuvant (CFA) containing 400 µg of *Mycobacterium tuberculosis* H37RA (DIFCO Laboratories, Detroit, MI), or 2) in incomplete Freund's aduvant (IFA) an emulsion containing various quantities (10 µg to 50 µg) of MSRV-Env, either as complete protein or as Env-SU part, or 3) in IFA alone. Immediately thereafter the mice received an intraperitoneal injection of *Bordetella pertussis* toxin (550 ng of in 0.1 mL of PBS per mouse). Mice were examined daily for at least 30 days for disability. Clinical scores were defined as follows: 0, no signs; 1, loss of tail tonicity or hyper-reflexia of hindlimb(s) or unilateral hindlimb weakness; 2, bilateral hindlimb or forelimb weakness; 3, plus unilateral paralysis or major deficit; 4, complete hindlimb or forelimb paralysis; 5, plus partial paralysis or major deficit of opposite limbs; 6, moribund or death. EAE induction experiments were consistently repeated in animal facilities of Lyon 1 University, Grenoble University and Hooke Laboratories in Lawrence (MA), following respective animal experimentation regulation with approval of local ethic committees for animal research.

### Histological analysis

At the termination of the experiment, spinal cords and brains were collected from two mice treated by Env protein which had developed clear signs of EAE (mice 1-1 and 1-2) and from one mouse which had significant weight loss during the first 2 weeks after immunization, but which never developed clinically detectable paralysis (mouse 2–5). Histological analysis was performed on these tissues.

After the mice were euthanized and perfused with PBS, the spinal cords and heads were collected into buffered formalin. For each mouse, 9 to 12 Luxol fast blue stained sections and 9 to 12 hematoxylin and eosin (H&E) stained sections, from lumbar, thoracic, and cervical spinal cord, were prepared and analyzed (3 to 4 sections from each region of the spinal cord). In addition, 3 Luxol fast blue stained sections and 3 H&E stained sections, from medulla oblongata, hind part of cerebellum, and rostral part of cerebellum, were prepared and analyzed from mice 1-1 and 1–2 (1 section from each region of the brain). Histological analysis was performed blind by a pathologist who was not aware of the clinical scores of the mice.

#### Information on microscope image acquisition

Make and model of microscope: Zeiss Imager A1; Type, magnification, and numerical aperture of the objective lenses: Zeiss Plan-NEOFLUAR 5×/0.16 Zeiss Plan-NEOFLUAR 40×/0.75; Imaging medium: air; Camera make and model: Zeiss AxioCam ERc 5s (with 0.5 adapter); Acquisition software: Zeiss AxioVision.

#### Count of inflammatory foci

Inflammatory foci of approximately 20 cells were counted in each H&E stained section. When inflammatory infiltrates consisted of more than 20 cells, an estimate was made of how many foci of 20 cells were present.

#### Estimation of demyelination

The demyelination score represents an estimate of the demyelinated area for each section as follows:

0 – no demyelination (less than 5% demyelinated area); 1–5 to 20% demyelinated area; 2–20 to 40% demyelinated area; 3–40 to 60% demyelinated area; 4–60 to 80% demyelinated area; 5–80 to 100% demyelinated area

For Luxol fast blue stained slides, the size of the demyelinated area was estimated based on less intense blue staining of myelin.

For H&E stained sections, the demyelinated area was estimated by looking for interruption of normal structure – pallor and vacuolation consistent with edema and demyelination, and dilated axons.

#### Count of apoptotic cells

The number of apoptotic cells in the H&E sections was determined.

### Myelin auto-antigen recall

At the end of the study period (day 30), mice from Env-SU series with clinical signs of EAE and controls were injected i.p. with 200 µg of MOG 35–55. Three days later, mice were sacrificed and spleen collected. Suspension of spleen cells were cultured for 24 h, 48 h and 72 h at 2×10^6^ cells/ml in RPMI 1640 (Invitrogen: Gibco 72400) supplemented with 20% FCS with or without addition of MOG (1 µg, 5 µg, 10 µg and 20 µg). In total, 5 independent culture experiments were performed, employing a total of 3 IFA mice and 6 Env mice.

## Results

In order to analyze the effects of the MSRV-Envelope protein under similar conditions to our previous study [31], we initially used the same surface subunit (Env-SU) that contains the TLR4-activating site. Thereafter, the entire envelope protein (Env) was used for a series of EAE studies in which detailed histological analysis was performed.

### MSRV-Env induced surface expression of dendritic cell maturation markers

**Figure 1:**
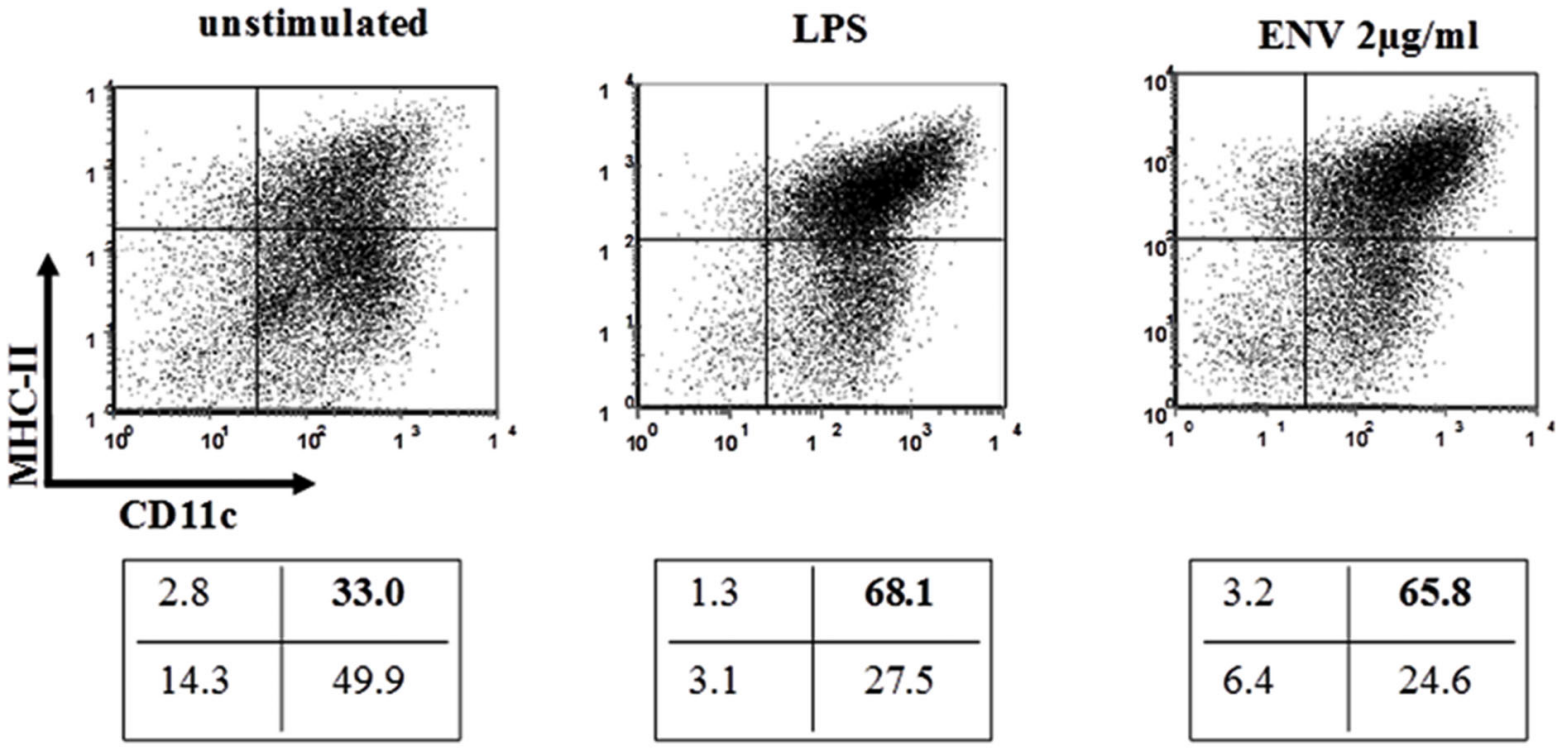
Env-SU induces the maturation of murine DC. DC from wild-type C57Bl/6 mice were stimulated with LPS (2 µg/mL) or Env-SU (2 µg/mL). After 24 h of incubation, cells were analysed by cytofluorometry for MHC class II and CD11c surface markers expression. DC were harvested, washed in PBS with 3% FCS and 0.16% sodium azide and immunostained for MHCII (monoclonal IA^b^-PE antibody) and for CD11c (monoclonal CD11c-APC antibody) for 30 min at 4°C. The conjugated Abs used for staining were all obtained from BD Biosciences. A propidium iodide staining has been used to exclude dead cells. Double positive cells for CD11c and MHC-II (upper right quadrant) represents mature DC. Upper panel: Graphic distribution of cells according to immunostaining intensity for CD11c (abscissa) and MHCII (ordinate) without stimulation (left), after stimulation by LPS (center) or after stimulation by MSRV-Env (right). Lower panel: Corresponding percentage of cells in each quadrant, below each graphic. Results are representative for three experiments.

To determine whether MSRV-Env-SU stimulated phenotypic changes associated with dendritic cell (DC) maturation, flow cytometry analyses were performed for the expression of CD11c and MHC class II (MHCII) by primary DC derived from bone marrow of mice. The CD11c marker is found on immature and mature DC and the MHCII molecules expression increase specifically on mature DC. Immature DC were treated with 2 µg/mL Env-SU, 2 µg/mL LipoPolySaccharide (LPS, a TLR4 pathway activator), or 20 µg/mL PSB (Pansorbin, a TLR2 pathway activator). After 24 h of incubation, DC maturation was measured by flow cytometry analysis for MHCII expression among CD11c positive cells (Figure 1). The proportion of double positives cells (MHCII^+^, CD11c^+^) among LPS or MSRV-Env-SU stimulated DC was strongly increased (68.1% and 65.8%, respectively) compared to unstimulated DC (33%). At the same time, there was a decrease in the number of CD11c^+^, MHCII^−^ cells (49.9% in unstimulated *versus* 27.5% and 24.6% in stimulated DC), indicating that DC differentiated to mature state. These results show that Env-SU, similar to LPS, can efficiently induce phenotypic maturation of DC from mice.

### MSRV-Env stimulates the production of pro-inflammatory cytokines in murine DC cultures

**Figure 2:**
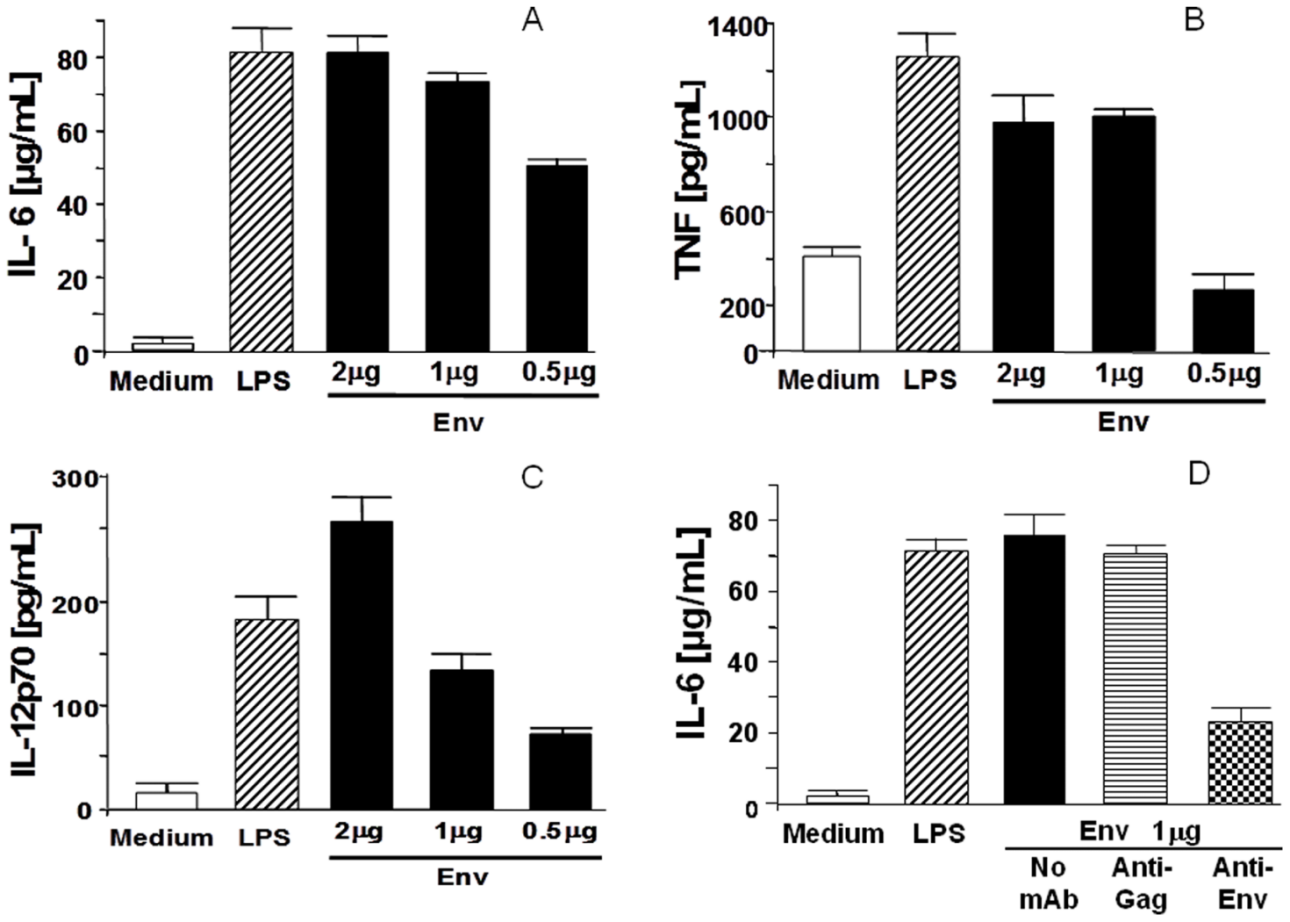
Env-SU induced the production of pro-inflammatory cytokine in DC culture. Murine Bone marrow DC were stimulated with LPS (2 µg/mL), PSB (100 µg/mL) and different amounts of Env-SU for 24 h. Culture supernatants were then analysed by ELISA for IL-6 (A), TNFα (B) and IL-12p70 (C) production. In (D), the specific inhibition of MSRV-Env stimulated DC by anti-Env neutralizing monoclonal antibody (GN-mAb_03) is shown, compared to an irrelevant anti-MSRV Gag (GN-mAb_12) or to diluents without antibody. IL-6 levels obtained in non-stimulated DC (medium) as negative control, or stimulated with LPS as positive control are also shown. IL-6 production was measured by ELISA in supernatants after 24 h of incubation (ELISA standard deviations <5%). These results are representative of three experiments realised under identical conditions.

To analyse pro-inflammatory cytokine production, we harvested and stimulated C57BL/6 DC with increasing concentrations of Env-SU for 24 h. Secretions of IL-6, TNFα and IL-12p70 in cell culture supernatants were analysed by ELISA (Figure 2). LPS stimulation was used as the reference for the TLR4 pathway activation. We observed that Env-SU induced the production of the pro-inflammatory cytokine IL-6 in a dose dependent manner, similarly to LPS (Figure 2A). In addition, IL-12p70 (Figure 2B) and TNFα (Figure 2C) production was also increased in a dose dependant manner, similarly to the production induced by LPS.

To assess the specificity of Env-SU pro-inflammatory properties, we studied the effects of anti-Env monoclonal antibodies (mAb) on the cytokine production (Figure 2D). We used a mAb targeting specific Env epitope, which was shown to neutralize Env-SU effects in human PBMC [31]. DC were incubated for 24 hours with Env-SU or LPS in the presence of mAb. DC showed a significant decrease of IL-6 production when our mAb was added in Env-SU stimulation, compared to DC incubated without mAb. No effect of this mAb was observed in LPS stimulation. IL-6 secretion was not affected in either case by treatment with an irrelevant control mAb specific for HERV-W Gag antigen (expressed from an MSRV clone).

In conclusion, our data demonstrate that MSRV-Env-SU induces the production of pro-inflammatory cytokines in a specific manner by C57Bl/6 DC that correlate with previous findings in human PBMC and DC (Rolland et al., 2006).

### Cytokine production by TLR4-KO and CD14-KO DC in response to MSRV-Env

**Figure 3:**
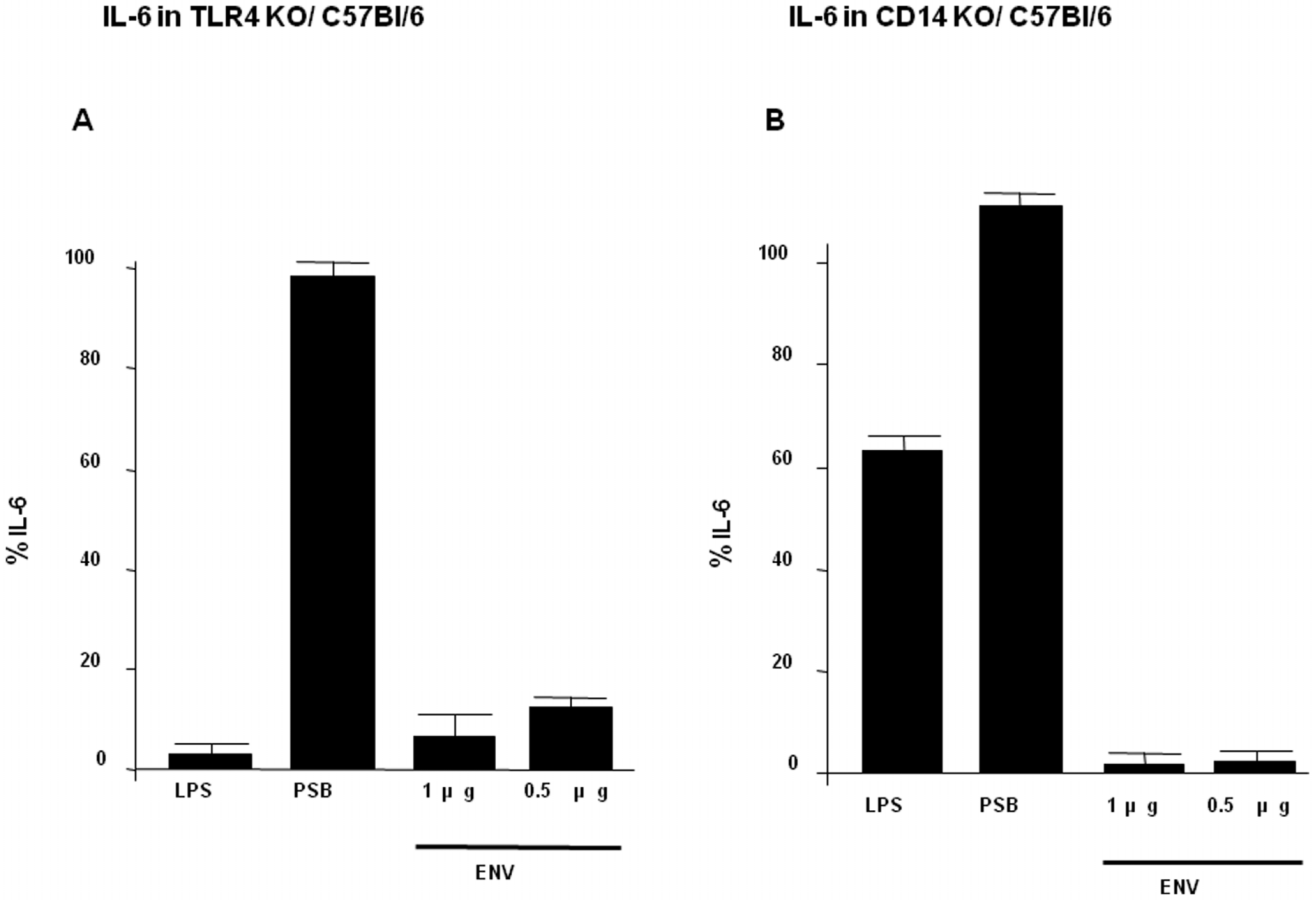
Comparison of IL6 production in Env-SU stimulated murine DC derived from TLR4 KO and CD14 KO mice compared with wild-type mice. IL-6 production in bone marrow DC culture supernatants of TLR4 KO (A) and CD14 KO (B) mice compared to wild-type C57BL/6 mice after 24 h of stimulation with LPS, PSB and graded dose of Env-SU. Percentages shown in the histogram correspond to the percentage of residual IL6 secretions dosed in cells supernatants of TLR4 or CD14 KO mice relative to wild-type mice. Data presented are representative of three experiments under identical conditions.

DC are professional antigen presenting cells expressing a variety of Toll-like receptors (TLR). TLR are essential in host defence against pathogens due to their capacity to detect microbes and initiate the innate immune response. We previously showed that TLR4/CD14 is triggered by MSRV-Env in human PBMC and DC (Rolland et al., 2006). Therefore, we checked whether this pathway is also engaged by this HERV agonist in mouse DC using TLR4 Knock Out (KO) and CD14 KO mice as the source of cells (Figure 3). LPS-induced production of IL-6 was significantly decreased in TLR4-KO mice compared to wild-type mice, whereas IL-6 production induced by pansorbin (PSB), targeting TLR2, remained the same for DC of both mice (Figure3A). Similar results were observed for CD14-KO mice DC although IL-6 secretion was less decreased in CD14-KO mice than in TLR4-KO mice, which can be explained by only partial dependency of LPS activation on CD14 (Figure 3B). These results confirmed that the TLR4 pathway of DC from TLR4-KO mice was functionnaly impaired in TLR4-KO mice and that the TLR2 pathway, induced by PSB, was still functioning as expected. As Env-SU failed to activate DC derived from either TLR4-KO or CD14-KO mice, MSRV-Env stimulation of murine DC was shown to depend on the TLR4 pathway in which the co-receptor CD14 participates as an accessory molecule.

### Immunization with MOG in presence of MSRV-Env induces EAE symptoms in C57Bl/6

**Figure 4:**
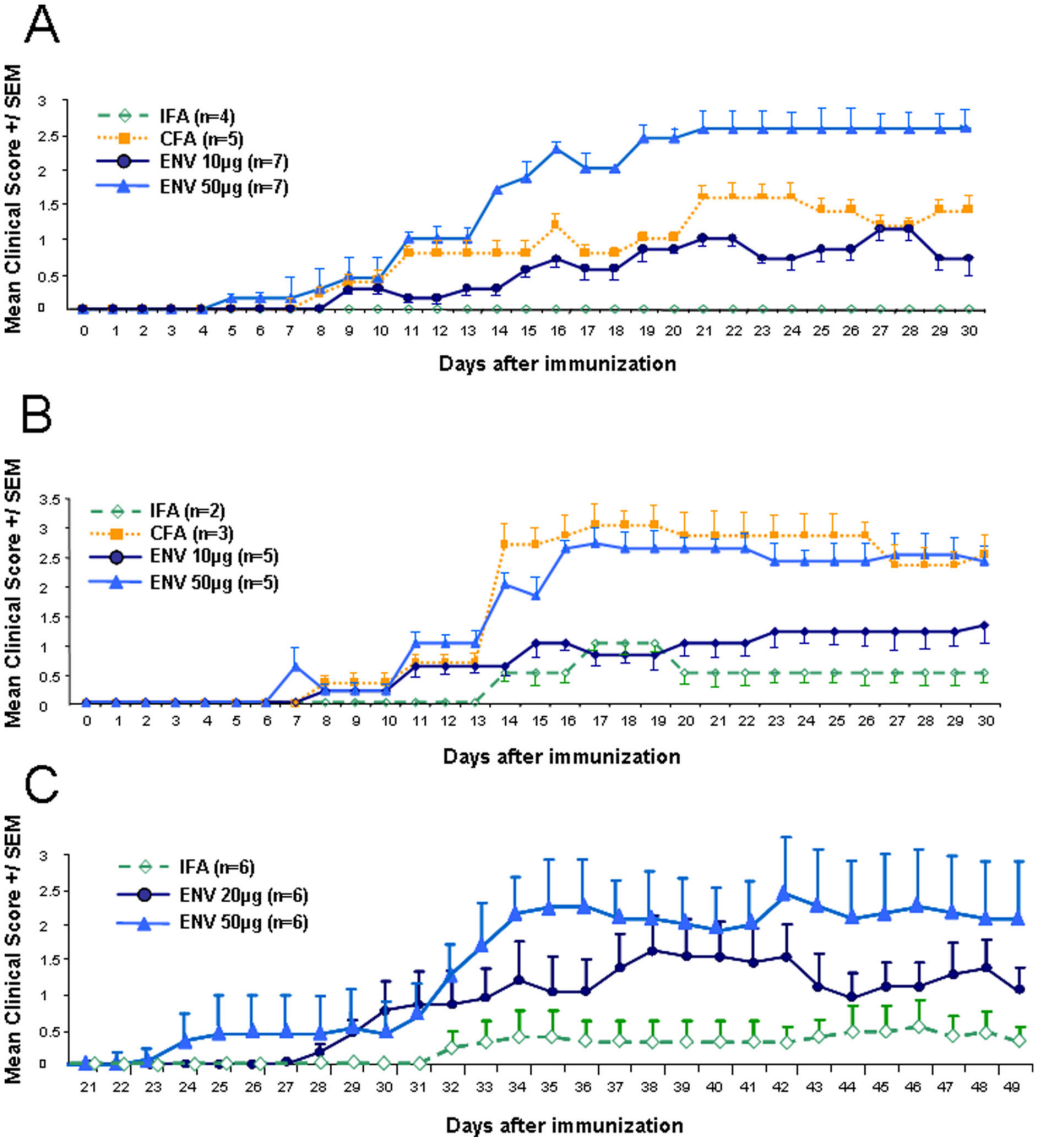
MSRV induced EAE like symptoms in C57Bl/6 mice. Experiments were performed on 6–8 weeks-old C57BL/6 female mice. Mice were immunized subcutaneously with 200 µg of MOG 35–55 per animal emulsified in CFA containing 400 µg of *Mycobacterium tuberculosis* H37RA, or an emulsion containing 10 µg or 50 µg of Env-SU in IFA, or an emulsion containing 20 µg or 50 µg of full-length Env protein (in C), or MOG with IFA alone. Immediately thereafter and again 2 days later, the mice received an intraperitoneal injection of 200 ng of *Bordetella pertussis* toxin in PBS. Mice of IFA, CFA or Env groups were examined daily and clinical score were then monitored on the clinical scale of 0 to 6. Mean scores and standard deviation (A–B) or SEM (C) for each group of mice are presented. The values are representative for three experiments with Env-SU and more than three with full-length MSRV-Env (with protocol variants) that repeatedly showed similar induction of EAE.

Experimental allergic encephalitis (EAE), the mouse model for MS, experimentally addresses the pathogenesis of the central nervous system injury following an autoimmune response (CNS). In order to study the potential role MSRV-Env may play in vivo, C57Bl/6 mice were immunized with myelin peptide MOG35–55 emulsified with either the complete Freund's adjuvant (CFA) or MSRV-Env-SU in incomplete Freund's adjuvant (IFA). In addition, as classically used in EAE for its ability to trigger the disruption of the blood brain barrier, Bordetella Pertussis Toxin (PTX) was injected as indicated in Material and Methods. Figure 4 shows the clinical scores of two different experiments (representing repeatedly obtained experimental series with Env-SU; n>3) between day 0 and day 30. Table I indicates the disease incidence and the mean clinical scores of the different groups (IFA, CFA, Env-SU 10 or 50 µg) of injected mice at different days. The scores represent the means of animals in each group. A period of latency between seven to ten days was noticed after immunization, followed by a progressive increase in the clinical score from day 10 to day 30 - in both Env-SU and CFA treated mice (Figure 4A&B). Moreover, as clinical scores were higher in the group injected with 50 µg than with 10 µg of Env-SU, the response appeared dose-dependent.

**Table 1:** Immunisation with MOG and MSRV-Env-SU induces dose-dependent EAE-like symptoms in C57BL/6 mice.

Group Treatment	disease	mean disease	mean score	max score	mean cumulative
	incidence (nb)	onset (d)	d30		score d30
IFA	0/4	-	0	0	0
CFA	5/5	12,2	1,4	1,8	31,2
ENV 50 µg	7/7	9,1	1,9	2,9	56,9
ENV 10 µg	7/7	14,6	0,4	1,6	18,7

Data correspond to those illustrated in figure 4A. Disease incidence, mean time to disease onset, mean clinical score at day 30, mean maximum score and mean cumulative score at day 30 were calculated for each group of mice.

Similar experiments of EAE induction were performed with the full-length MSRV-Env protein in C57/BL6 mice, as its synthesis and purification became available (Cf. Figure S2, S3 in File S1) and because multiple epitopes compatible with full-length Env protein have been detected in MS brain lesions [26]. MOG-EAE induction with the full-length Env protein in C57/BL6 mice was also reproduced in a reference laboratory (Hook laboratories, MA, USA) as presented here with two doses (20 µg and 50 µg) over a 49 days study period (Figure 4C).

As illustrated in Figure 4C no mice showed signs of paralysis up to Day 21, which appears to be a longer delay that what was generally observed with Env-SU. Between Days 22–31 (50 µg) or 22–33 (20 µg) mild signs of paralysis appeared in several mice. On Days 31–33 (50 µg) and 34–37 (20 µg), mice showed clear onset of paralysis that was indistinguishable from paralysis which develops in standard MOG35–55-induced EAE (Figure 4C). The disease progressed further, and by Day 49, 5 of 6 mice (50 µg) and 6 of 6 mice (20 µg) had developed EAE. The mean maximum score (MMS), the mean day of onset and the weight gain at the termination of the study (in %, relative to the weight measured on Day-1), are presented in Table II. Statistical analyses are also presented in Table II and confirm the significant differences between the control group (IFA+MOG) and the mice injected with Env. Interestingly, with similar numbers tested in this experiment (n = 6) the MMS value becomes significantly different from the control group in mice injected with 50 µg of Env protein (p<0.01), whereas it is at the limit of significance (p = 0.58) in mice injected with 20 µg. This indicates a dose-dependent effect on the neurological symptoms, whereas the impact on the weight kinetics appears similar with the two MSRV-Env doses.

**Table 2:** Statistical Analysis of EAE-like symptoms after Immunisation with MOG and MSRV-Env complete in C57BL/6 mice.

Group Treatment	Day of Onset +/− SD	p value Time to onset	MMS +/− SD	p value MMS	% body weight at end of experiment +/− SD	p value body weight	Died during study
No Env	37.0+/−7.1		0.92+/−1.24		120.1+/−8.3		0/6
Env 20 µg	31.7+/−4.0	0,0078*	2.25+/−0.94	0,0581	110.2+/−8.5	0,0339*	0/6
Env 50 µg	29.2+/−3.6	0,0198*	2.92+/−1.69	0,0398*	101.7+/−18.9	0,0323*	1/6

Data correspond to those illustrated in figure 4C. Time to onset was compared using Wilcoxon's survival test; MMSs were compared using Wilcoxon's non-parametric test; Change in body weight at the end of the study was compared using Student's t-test.

^*^ Statistically significant value.

Altogether, these data demonstrate that immunization with MOG35–55, together with either Env-SU or entire Env protein of MSRV is able to induce in vivo neuropathological inflammation indistinguishable from classic EAE.

### Histological analysis

**Figure 5:**
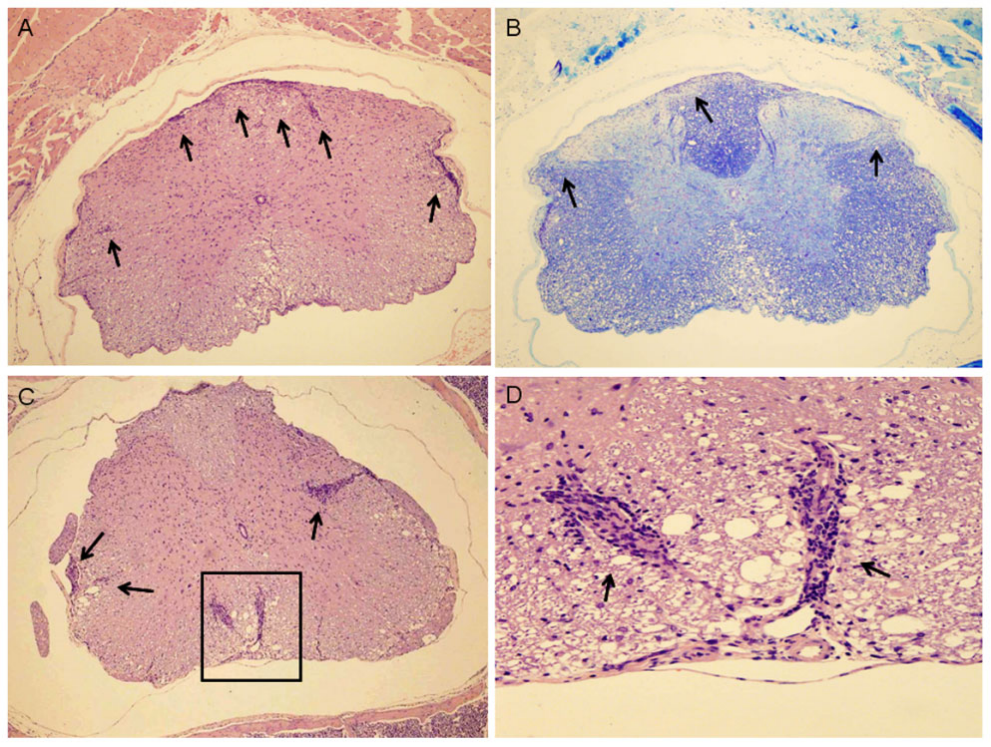
Histological analysis of cervical spinal cord (mouse #1-1). A. Microscopic changes in transverse section of spinal cord are shown (100×). Multiple inflammatory foci and some multifocal inflammatory lesions are present in the leptomeninges, around blood vessels in the leptomeninges and white matter, and parenchyma of the white matter (arrows). There is also vacuolation in the white matter that is consistent with edema and demyelination. B. Luxol fast blue stained section of cervical spinal cord (100×). This section is from the same block of tissue as the H&E stained section shown in A. Areas of demyelination are visible within white matter (lighter blue stained areas, in the outer parts of the spinal cord; arrows). C. H&E stained transverse section of thoracic spinal cord (100×). Four multifocal inflammatory lesions are present in the leptomeninges, around blood vessels in the leptomeninges and white matter (box and arrows). There also is vacuolation in the white matter that is consistent with edema and demyelination (box). D. H&E stained section of thoracic spinal cord (400×). Detail of the above slide (boxed area). Two multifocal inflammatory lesions are shown (arrows).

Histological analysis confirmed the clinical data of MSRV-Env induced EAE. Mice 1-1 and 1-2 with MMS of 3.5 and respective clinical scores of 2.5 and 3.5 at the end of the study were examined (Table I, and details in section II of File S1). Analysis was performed on 3 regions of the spinal cord and 3 regions of the brain. Between 4 and 7 inflammatory foci were found in each analyzed section of the spinal cords of both mice (Figure 5A, C–D). Mild to moderate demyelination (scores 1 to 3) was found in each analyzed section of the spinal cord stained with Luxol fast blue (Figure 5B). Demyelination scores were similar for the H&E and Luxol fast blue stained sections. Between 1 and 5 apoptotic cells were found in each analyzed section of the spinal cord (Cf. Table S5 in File S1). This is similar to what is seen in standard MOG35–55/CFA-induced EAE and is not observed in IFA controls (data not shown).

Demyelination is considered difficult to estimate in brain sections of mice with EAE, demyelination levels are usually not assessed in brains of mice with standard MOG35–55-induced EAE. Since this MSRV-Env model is novel, an attempt was nonetheless made to estimate the demyelination in the brain sections. Between 3 and 8 inflammatory foci were found in each analyzed section for both mice. In the Luxol fast blue stained sections, demyelination score was 1 to 3 in areas of inflammation. In H&E stained sections we estimated the percent of inflamed areas with interruption of normal structure, with pallor and vacuolation consistent with edema and demyelination (Cf. Figures S10 to S21 in File S1). Apoptotic cells were also found in 4 of 6 of these sections (4 to 9 apoptotic cells/section).

Inflammatory infiltrates consisted predominantly of mononuclear cells. Demyelination was detected in both H&E and Luxol fast blue stained sections. The number of inflammatory foci, the number of apoptotic cells and the severity of demyelination were consistent with the clinical scores (for detailed Tables, see section II and annexed raw data of File S1).

Such histological aspects of the lesions in the spinal cords and brains of Env-induced EAE mice were not different from the findings typical of standard EAE. In addition, brain lesions with marked demyelination were readily detectable in MSRV-Env induced EAE.

Similar histological analysis was performed on an additional case, mouse 2–5, which had significant weight loss during the first 2 weeks after immunization but which did not develop clinically detectable paralysis during the study period, in order to address potential lesions in a sub-clinical phase of EAE.

Three regions of the spinal cord were analyzed in parallel with the above described mice 1-1and 1-2 (Figure 5), and 3 sections from each region were prepared and scored. Mouse 2–5 had inflammatory foci in all 3 sections of the lumbar region (1 to 2 inflammatory foci/section), but none in the thoracic or cervical regions. Apoptotic cells were found in 16 out of the 18 sections examined from mice 1-1 and 1-2, but none were found in the 9 sections examined from mouse 2–5 (details in section II of File S1).

These finding are consistent with a mild inflammation of the spinal cord in that could explain the absence of clinically detectable signs despite an ongoing lesion process in several animals of our EAE group.

### MOG recall of immunized mice leads to γIFN production

**Figure 6:**
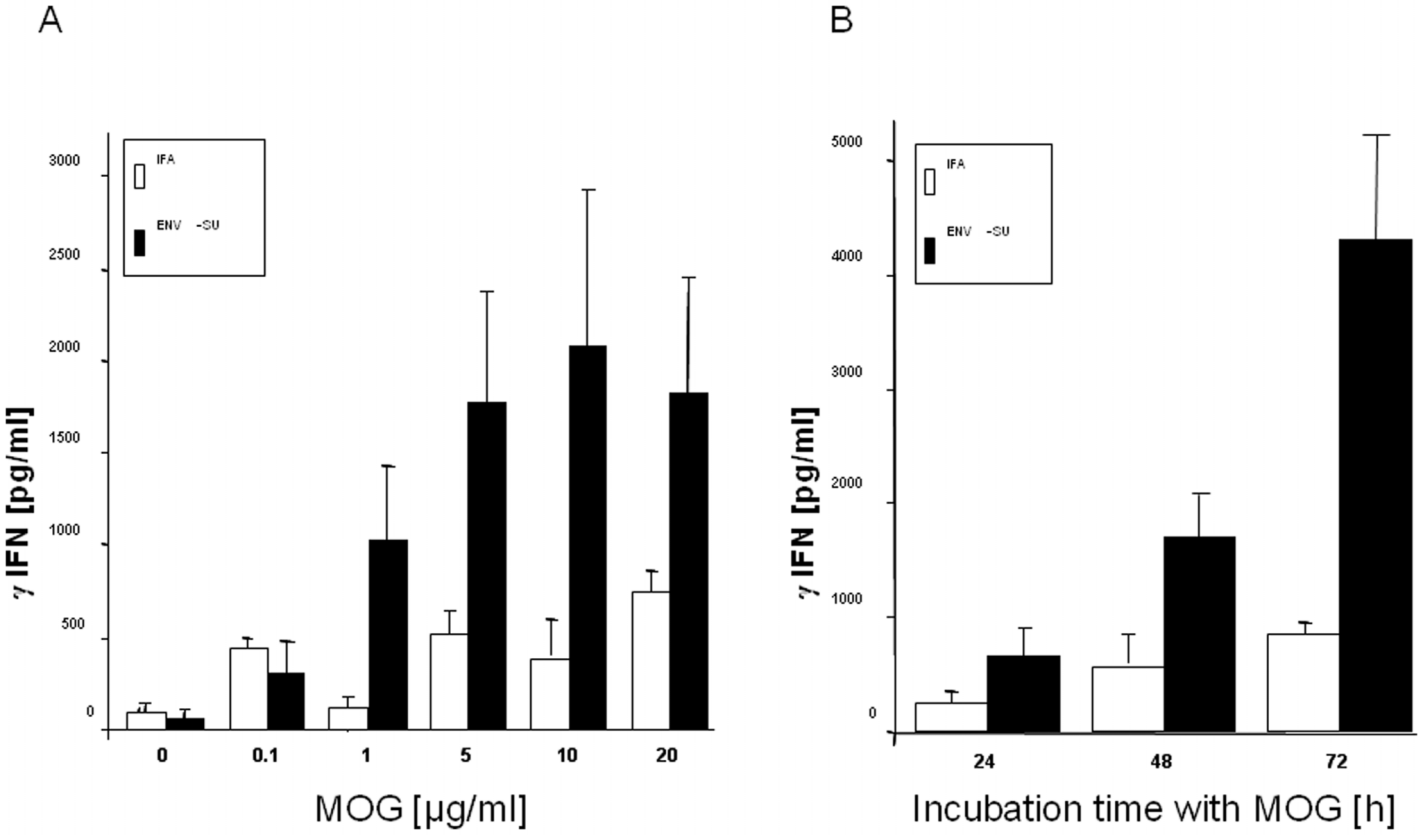
EAE mice induced by Env-SU display anti-MOG autoimmunity in splenocytes cultures. A. γIFN secretion of pooled splenocytes (2 mice for the IFA group and 3 mice for the Env-SU group) of EAE mice assessed by ELISA of culture supernatants 24 h after stimulation. B. Kinetics of γIFN production upon MOG recall on pooled splenocytes of IFA (n = 3) and Env-SU (n = 3) injected EAE mice. IFN-γ secretion is assessed in culture supernatants after 24 h, 48 h and 72 h of incubation. Results are representative of two experiments.

In order to investigate a specific immune response toward the MOG35–55 antigen, splenocytes were harvested from MOG35–55/IFA or MOG35–55/Env-SU immunized mice, three days after recall injection of MOG on day 30 (Cf. Materials and Methods). Cultured T lymphocytes from spleen of MOG35–55/Env-SU immunized mice responded to MOG35–55 challenge with a significant secretion of γIFN, which was not observed in the cultured splenocytes from MOG35–55/IFA immunized control animals (Figure 6A). T cells from Env-SU treated mice produced significant amounts of γIFN in a dose dependent manner to reach a maximum at 10 µg/mL of MOG35–55. Kinetic analysis of the anti-MOG T-cell response showed significantly elevated γIFN productions at 24 h (800 pg/mL), 48 h (1800 pg/mL) and 72 h (4200 pg/mL) in Env-SU mice when compared to -IFA treated control mice (Figure 6B).

These results ascertain that the combination of MOG35–55 antigen with MSRV-Env-SU, is able to promote specific autoimmune response towards this antigen with the characteristics of a Th1 polarisation of T lymphocytes similar to the features of classic EAE.

## Discussion

The envelope protein expressed by a retroviral element of the HERV-W family, MSRV [12], was previously shown to display potent pro-inflammatory activity either when associated with virion particles, or as a full-length protein (MSRV-Env), or as the fragment encompassing its surface subunit (Env-SU) [30], [31]. Previous studies have shown that MSRV particles exert potent pro-inflammatory effects *in vitro*, in human PBMC and DC (Rolland et al., 2006) as well as *in vivo*, in humanized SCID mouse model grafted with human lymphoid cells, in which injected MSRV virions caused an overexpression of TNF-α leading to death by brain haemorrhages [30], [33]. Furthermore, MSRV-Env-SU was found to provoke this inflammatory reaction through engagement of the TLR4/CD14 pathway [31]. The present study reveals that MSRV-Env is also able to activate cells of the innate immune system in mice leading to proinflammatory cytokine productions through pattern recognition receptors TLR4 and CD14. Furthermore, when associated with MOG antigen, specific immune response of T cells producing γ-interferon is mounted. These combined innate and specific acquired immune responses lead to the development of neuropathology similar to standard EAE.

TLR constitutes a large family of receptors which is characterized, among other features, by the nature of their ligands. TLR are expressed in mammalian immune cell types such as B cells [34], macrophages, monocytes, dendritic cells [35], oligodendrocytes [36], astrocytes [37], microglia [38] and neurons [39]. TLR can sense distinct microbial products and are central to innate immune response to various classes of pathogens [40], [41]. Among viruses known to activate TLR, an endogenous retrovirus such as Mouse Mammary Tumor Virus (MMTV) has been found to mediate activation and cellular maturation of murine DC *in vitro* and *in vivo* by direct interaction with TLR4 [42]. Moreover, specific activation of CNS innate immunity through TLR4 can lead to neurodegenerative processes via activation of brain-resident macrophages [43] and TLR4 is necessary for LPS-induced oligodendrocyte injury in the CNS [44]. These results indicate that the engagement of TLR4 receptor expressed on CNS resident, and/or perivascular, macrophages by its ligands can contribute to oligodendrocyte damage and neurodegeneration.

By inducing the production of proinflammatory cytokines by murine immune cells via CD14 and TLR4 pathway, MSRV-Env can thus cause neuroinflammation and brain damage. Its presence in MS plaques also suggested that MSRV-Env is a relevant candidate capable of causing the immunopathogenic features of human neuroinflammatory diseases such as MS [26]. The pro-inflammatory effects of MSRV-Env have been shown to result from TLR4 and CD14 receptor pathway activation, because TLR4 blockade strongly inhibited the induction of the T-cell specific cytokine (γIFN) by MSRV-Env protein, in human PBMC cultures [31]. In the present study, we showed that MSRV-Env activation of DC is completely abrogated in mice deficient for either TLR4 or CD14. Thus, the pro-inflammatory properties of MSRV-Env protein from HERV-W appear to involve the activation cascades initiated by the engagement of CD14 and TLR4 in human and mouse cells as well.

In the central nervous system, TLR are expressed on different cell types and can influence local immune responses. For example, there is a marked increase in the expression of TLR within MS brain lesions or cerebrospinal fluid mononuclear cells, as well as within demyelinated lesions of EAE [36]. Thus, EAE has proven invaluable in understanding distinct clinical and pathological features of MS [45], [46], [47]. In the present study, we have therefore addressed the potential of MSRV-Env protein to trigger the development of EAE. Our data demonstrate that mice injected with MSRV-Env in the presence of MOG 35–55 peptide clearly developed EAE. Demyelinating neuroinflammation in the CNS was confirmed by neurohistological analysis. In addition, significant inflammatory and autoimmune responses against the MOG35–55 peptide were revealed. The body weight changes suggest that this new model of EAE may be remitting-relapsing, with the first wave of EAE being subclinical. Similar clinical observations are often made in SJL mice, in which some mice develop a very mild, barely detectable first wave of EAE (with only a single day of clinically detectable paralysis, with a score of 0.5), but go on to develop a significant relapse 10–15 days later, with a score of 2.0 or greater [6], [9]. The body weight changes and histological analysis suggest that in addition to the mice with clear, clinical signs of EAE, additional mice developed CNS inflammation which was not detected in the clinical examination during the time limits of the present study.

These *in vivo* observations show that the mycobacterial extract of CFA, used in standard EAE protocol, can be replaced with the purified recombinant MSRV-Env or Env-SU protein providing adjuvant effects necessary for EAE induction in C57/Bl6 mice. Importantly, the mycobacterial lysate present in CFA appears irrelevant for studying upstream inducers of neuroinflammation in MS since mycobacteria are not present in MS lesions. Therefore, MSRV-Env EAE now offers a new *in vivo* model for further exploring the effect of experimental disease induction by this HERV-W protein and its potential role in MS as an upstream inducer of neuroinflammation. This is justified by the facts that the retroviral envelope protein from HERV-W endogenous retrovirus family (i) has been regularly detected in MS brain lesions [20], [25], [26], [32], (ii) can be abnormally expressed upon HERV-W activation by certain environmental viruses in permissive cells [48], [49] and (iii) is now shown to trigger *in vivo* the pathogenic features characterizing MS lesions.

Altogether, these data suggest that MSRV-Env expression in perivascular macrophages and/or microgliocytes [26], could be the missing-link between triggering environmental co-factors and the immunopathogenic cascades leading to the MS lesions and to disease progression. Interestingly, as an endogenous retrovirus gene, it involves a genetic interface between various infectious agents and the pathophysiological processes activated downstream by the engagement of TLR4 in immune cells, as well as in glial cells [50]. The consistency of such observations with MS pathophysiology leads to now ongoing clinical trials with a humanized antibody neutralizing the TLR4 agonistic effects of MSRV-Env protein in MS [51], [52].

In conclusion, our study proposes that MSRV-Env could be the common link between various triggering co-factors (e.g. infections with an Herpesvirus such as EBV) and the immunopathogenic cascade leading to neuroinflammatory diseases such as MS. Although phylogenic analyses have shown that HERV-W is only present in human and in old world monkeys (Sverdlov et al., 2000), our data demonstrate that mice are susceptible to neuroinflammatory disease triggered with MSRV-Env, and can be used for studying MSRV-Env pro-inflammatory properties and therapeutic molecules targeting this HERV-W protein.

## Supporting Information

File S1 **Contains Supporting Information with Tables and Figures.**
(DOCX)
