# Supplementary material for: Human Endogenous Retrovirus Protein Activates Innate Immunity and Promotes Experimental Allergic Encephalomyelitis in Mice
Source: PLoS One. 2013 Dec 6;8(12):e80128. doi: 10.1371/journal.pone.0080128 (PMC3855614; doi:10.1371/journal.pone.0080128)
Supplement: File S1 — Contains Supporting Information with Tables and Figures. (DOCX) [file pone.0080128.s001.docx]

**FILE S1: Supporting Information**

**MSRV (HERV-W) ENVELOPE PROTEIN ACTIVATES**

**INNATE IMMUNITY AND CAUSES EAE Model**

**of Multiple Sclerosis IN MICE**

**Table of Content**

[**I. Independent and repeated experiences: Dose-response effect of Full-Length MSRV-Env protein in the MOG EAE model with C57BL/6 mice (Internal Feasability study).** 2](#_Toc369197158)

[**I.1 Material and methods** 2](#_Toc369197159)

[**I.2 Results** 5](#_Toc369197160)

[**I.3 Conclusions** 7](#_Toc369197161)

[**II. Independent and repeated experiences: EAE Studies in C57BL/6 mice (Complementary data to those presented in main manuscrip):** 8](#_Toc369197162)

[**II .1 Results** 8](#_Toc369197163)

[**II.1.a Changes in body weight and development of paralysis** 8](#_Toc369197164)

[**II.1.bResults of Histological analysis (Supporting data and Tables).** 9](#_Toc369197165)

[**II.2 Additional Figures to Figure 6 of the main manuscript (numbered from #7):** 13](#_Toc369197166)

[**II.3 Appendix (raw data).** 19](#_Toc369197167)

[**III. Recombinant envelope protein:** 21](#_Toc369197168)

[**III.1: Origin and structure:** 21](#_Toc369197169)

[**III.2. QC reports on production and purification of endofree recombinant MSRV-Env protein.** 22](#_Toc369197170)

[**III.1.a Env-SU:** 22](#_Toc369197171)

[**III.1.b Full Length MSRV-Env (Env-T):** 24](#_Toc369197172)

[Imprint date: January, the 5th 2011 24](#_Toc369197173)

**I. Independent and repeated experiences: Dose-response effect of Full-Length MSRV-Env protein in the MOG EAE model with C57BL/6 mice (Internal Feasability study).**

**I.1 Material and methods**

a) Mice

Pathogen-free female C57BL/6 mice (10 week-old) were purchased from the French commercial breeder Charles River. Animals were maintained 4/5 per cage on a standard light-dark cycle with ad libitum access to food and water and were undisturbed for an 8-days period of acclimation. Special care was taken to ensure very clean housing conditions. Particularly, animals were housed in special cages equipped with filter lid. Experiments were not GLP compliant but were performed in accordance with French and European Economic Community guidelines (86/09/EEC–Council Directive of 24 November 1986) for the care and use of laboratory animals. All efforts were made to minimize the number of animals used and their suffering.

b) Experimental Allergic Encephalomyelitis

| **Group** | **MOG** | **ENV (µg)** | **Diluents** | **PTX** |
| --- | --- | --- | --- | --- |
| **1** | + | 25 | IFA | + |
| **2** | + | 50 | IFA | + |
| **3** | + | - | CFA | + |
| **4** | - | 50 | IFA | + |
| **5** | + | - | IFA | + |
| **6** | + | - | IFA | - |
| **7** | + | - | PBS | + |
| **8** | - | - | IFA | + |
| **9** | - | - | PBS | + |
| **10** | - | - | PBS | - |

Mice were identified by tagging the tail or the back of each mouse and ten experimental groups of 3 mice were formed. The table S1 shows the composition of the solutions that have been injected to each group.

*Table S1: Composition of the solutions injected in each experimental group.*

*Preparation of immunogenic solutions*

The diluent was used volume / volume with the quantity of proteins. The mixtures were very well homogenized before injection using two syringes connected through a luer fitting and a 3-way valve. The desired amount of antigen solution was taken into a glass syringe. The volume should not fill more than half the syringe. An equal volume of the diluent is taken into another glass syringe. All air was removed and the syringes were connected through the luer fitting to the 3-way valve. The 3-way valve was adjusted such that the connection was open between the two syringes. The plunger was carefully depressed from the antigen solution first, pushing the antigen into the diluent. The plungers are alternately pushed, mixing the diluent and the antigen solution into an emulsion. This process was continued until the plungers were difficult to push. The 3-way valve was adjusted such that the connection towards outside was open. The emulsions were collected in a glass tube to avoid any wall adherence

*Injections of solutions*


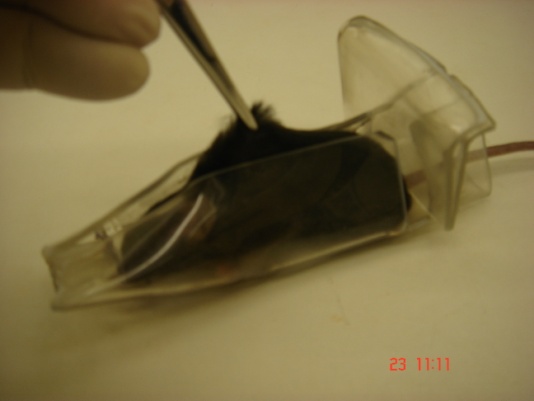
The immunogenic solutions were injected using a glass syringe with a 25 gauge needle to avoid any wall adherence. For each injection, mice were subcutaneously injected in the cervical neck, after disinfection of the sites of injection, for a localized reaction, closer to the CNS. It should be noted that a precise subcutaneous injection preventing stress and eventual product leaking outside from the injected mice was made possible with an in-house simple restrainer device conceived and made by Reza Firouzi (Figure S1). The PTX solution was intraperitoneally administered using a plastic syringe with a 25 gauge needle.

*Figure S1 – Photograph of the restrainer device conceived and made by Reza Firouzi for subcutaneous injections in the neck.*

*Timeline of the injections*

Mice were first immunized at D0 and boosted later twice at D7 and D14. In addition to the immunization mixture, some experimental groups received injections of PTX the same day of the immunization at D0, D7 and D14 and two days after at D2, D9 and D16.

c) Clinical monitoring and evaluation of symptoms

The clinical analysis carried out in this experiment was an assessment of the general locomotion with a rough six-steps scoring system. The visual evaluation of symptoms was regularly done (every two or three days) according to the following clinical score scale:

0 = No signs.

1 = Hyperreflexia of the hind limb(s) or unilateral hind limb weakness.

2 = Bilateral hind limb or forelimb weakness.

3 = Plus unilateral major paralysis or major deficit.

4 = Complete hind limb or fore limb paralysis.

5 = Plus partial paralysis or major deficit of opposite limbs.

6 = Moribund or dead.

In addition to the clinical score, the clinical monitoring included an assessment of the body weight of mice in order to establish a correlation between the course of the disease in terms of symptoms and the body weight of the mice.

From the beginning of paresis or paralysis signs, easily available hydrated food was given to the mice. For ethical issues concerning the pain management, the mice were sacrificed when the clinical score was more than 5.

**I.2 Results**

Because of the small size of the samples used in this study, standard deviation was not considered as relevant to examine the variability of the results in each group and only the means were represented in Figure S2 for the clinical scores and in Figure S3 for the body weight variation. For clarity, the body weight is expressed for each point as the body weight variation compared to the body weight measured at D0.

As already described in our previous studies (see report October – December 2009), all the mice of the EAE-groups (1, 2 and 3) developed clinical signs. No apparent neurological deficits could be observed before the second immunization. From D7, clinical score began to increase with the emergence of a light tail weakness evolving towards partial paralysis of hind limbs. After the third immunization at D14, a regular progression of the neurological deficits was observed and the clinical score increased up to 4 with mice exhibiting complete hind limbs paralysis.

Interestingly, mice of the groups injected with the MSRV-ENV protein (Groups 1 and 2) followed a very similar pattern of neurological deficits to that of the mice injected with MOG + CFA (Group 3). However, the clinical score observed after the last immunization was increased in animals injected with 50 µg of MSRV-ENV protein and in the control positive group compared to animals injected with 25 µg of MSRV-ENV protein. It is important that all mice injected with 50 µg of MSRV-ENV protein died before the end of the experiment, while only one mouse per group died in the other EAE-groups.

Moreover, none of the others groups exhibited any sign of neurological deficits during the experiment, except two mice of the group 4 (ENV 50 µg + IFA + PTX) that displayed a general weakness associated with the emergence of a bristling coat. This result is not really surprising because ENV-MSRV is known for its strong capacity to stimulate the immune system. It is very likely that, for these mice, a severe immune reaction induced by MSRV-ENV protein and exacerbated by the PTX could explain the deterioration of their general health status.

As shown in Figure S3, mice of the negative control groups showed a regular increase of the body weight during all the experiment. Conversely, control positive mice showed an atypical profile of body weight variation, probably due to the small size of the group. Interestingly, mice injected with both doses of MSRV-ENV protein displayed a very slight increase of the body weight from D0 to D14 and a decrease after the last immunization (D14). Strikingly, this decrease was more pronounced in mice injected with 50 µg of MSRV-ENV protein.

*Figure S2 – Mean clinical scores of each group during the experiment*

*Figure S3 – Mean body weight variation of each group during the experiment*

**I.3 Conclusions**

Our data suggest that the dose of 25 µg of MSRV-ENV protein is not sufficient to reproduce the classical clinical symptoms of an EAE animal model, while the dose of 50 µg is able to induce an experimental model for severe and acute MS-like episode and revealing quite more challenging for assaying therapeutic efficiency. The concomitance between the worsening of the clinical symptoms and the decrease of the body weight in ENV-MSRV EAE groups is highly indicative of the pathogenic potential of MSRV-Env in such animal models for studying MS.

Interestingly, we did not report emergence of any neurological signs in all the negative control groups. These data suggest that EAE is strictly induced in specific experimental conditions and that our data cannot be explained by side effects of one of the reagents used for the induction of EAE.

**II. Independent and repeated experiences: EAE Studies in C57BL/6 mice (Complementary data to those presented in main manuscrip):**

**II .1 Results**

**II.1.a Changes in body weight and development of paralysis**

In this experiment, the average body weight of the mice followed a cyclic pattern - increase on Days 1 and 3, then decrease between Days 6 and 13, then increase again between Days 15 and 29, after which point it decreased again (Figure S4; raw data in Appendix, Tables S.A1 and S.A2). The body weight changes as seen here are not expected in healthy mice. Seven of the 10 mice had one or more days when their body weight was at least 5% below their weight on Day -1. The first wave of body weight loss resembled weight loss observed during the course of standard MOG_35-55_-induced EAE, but no signs of paralysis were observed during this period. It is possible that the mice suffered a subclinical CNS inflammation during this period, which was the cause of weight loss.

**Figure S4. Changes in body weight over time**

85%

95%

105%

-2

3

8

13

18

23

28

33

38

43

48

**Day After Immunization**


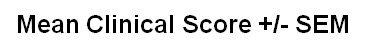


Up to Day 21, no mice showed signs of paralysis. Between Days 22 and 25, very mild signs of paralysis appeared in several mice (Figure S5; raw data in Appendix, Table S4). On Days 26 and 27, some mice showed clear onset of paralysis that was indistinguishable from paralysis which develops in standard MOG_35-55_-induced EAE. The disease progressed further, and by Day 46, 5 of 10 mice had developed EAE, with a mean maximum score (MMS) of 3.1 +/- 0.9 for the 5 mice which developed EAE. The MMS of all the mice in the group (including the mice with no signs of EAE) was 1.6 +/- 1.7. The average day of disease onset for the mice which developed EAE was 27.2 +/- 3.5 days after the first immunization. The average EAE score on Day 46 was 2.3 +/- 1.1 for the 5 mice which developed EAE and 1.2 +/- 1.4 for all 10 mice in the group (Table A3).

**Figure S5. Mean EAE score over time (Full-length ENV protein)**

**II.1.bResults of Histological analysis (Supporting data and Tables).**

Initial histological analysis was performed on 3 regions of the spinal cord and 3 regions of the brain, 1 section from each region from both mice 1-1 and 1-2. Between 4 and 7 inflammatory foci were found in each analyzed section of the spinal cords of mice 1-1 and 1-2 (Table S2).

Table S2. Count of inflammatory foci - initial histological analysis of spinal cords.

Mild to moderate demyelination (scores 1 to 3) was found in each analyzed section of the spinal cord stained with luxol fast blue (Table 2).

Table S3. Demyelination scores (Luxol fast blue sections) initial histological analysis of spinal cords.

Demyelination scores were similar for the H&E and luxol fast blue stained slides (Tables 2 and 3). Demyelination scores were similar to what would be expected in standard MOG_35-55_/CFA-induced EAE.

Table S4. Demyelination scores (H&E sections) initial histological analysis of spinal cords.

Between 1 and 5 apoptotic cells were found in each analyzed section of the spinal cord (Table S5).

Table S5. Count of apoptotic cells – initial histological analysis of spinal cords.

Between 3 and 8 inflammatory foci were found in each analyzed section of the brains of mice 1-1 and 1-2, (Table S6).

Table S6. Count of inflammatory foci - histological analysis of brains.

In the luxol fast blue stained sections, demyelination score was 1 to 3 in areas of inflammation (Table S7).

Table S7. Demyelination scores (Luxol fast blue sections) - histological analysis of brains.

In H&E stained sections we estimated the percent of inflamed areas with interruption of normal structure, with pallor and vacuolation consistent with edema and demyelination (Table S8).

Table S8. Demyelination scores (H&E sections) - histological analysis of brains.

Apoptotic cells were found in 4 of 6 of these sections (4 to 9 apoptotic cells/section; Table S9).

Table S9. Count of apoptotic cells – histological analysis of brains.

Additional histological analysis was then performed on the spinal cords from the two mice initially analyzed (1-1 and 1-2) and from mouse 2-5, without clinically detectable paralysis during the study period. Three regions of the spinal cord of these 3 mice were analyzed, and 3 sections from each region were prepared and scored. Inflammatory foci were found in all the analyzed sections of the spinal cords from mice 1-1 and 1-2 (Table S10, Figures S11, S13, S14, S15 and S17).

Table S10. Count of inflammatory foci - histological analysis of spinal cords.

Mouse 2-5 had inflammatory foci in all 3 sections of the lumbar region (1 to 2 inflammatory foci/section), but none in the thoracic or cervical regions (Table S10, Figure S20). This finding is consistent with a mild inflammation of the spinal cord that may not be clinically detectable.

Demyelination was detected in all sections of all the regions of the spinal cords from mice 1-1 and 1-2, but only mild demyelination (score 1) was found in one of the three sections of the lumbar region, and no demyelination was found in the other two regions of the spinal cord from mouse 2-5 (Tables S11 and S12, additional Figures S10, S12, S14, S17, S19 and S21). This is consistent with the clinical observations of these mice.

Table S11 Demyelination scores (Luxol fast blue sections) - histological analysis of spinal cords.

Table S12. Demyelination scores (H&E sections) - histological analysis of spinal cords.

Apoptotic cells were found in 16 out of the 18 sections examined from mice 1-1 and 1-2, but none were found in the 9 sections examined from mouse 2-5 (Table 12).

Table S13. Count of apoptotic cells - histological analysis of spinal cords

.

**II.2 Additional Figures to Figure 6 of the main manuscript (numbered from #7):**

**Figure S10.** **Luxol fast blue stained section of thoracic spinal cord, mouse #1-1 (100x)**

This section is adjacent to the H&E stained section shown in Figure 6C of the main manuscript. Areas of demyelination are visible within white matter (lighter blue stained areas, in the outer parts of the spinal cord; examples in boxed areas).

**
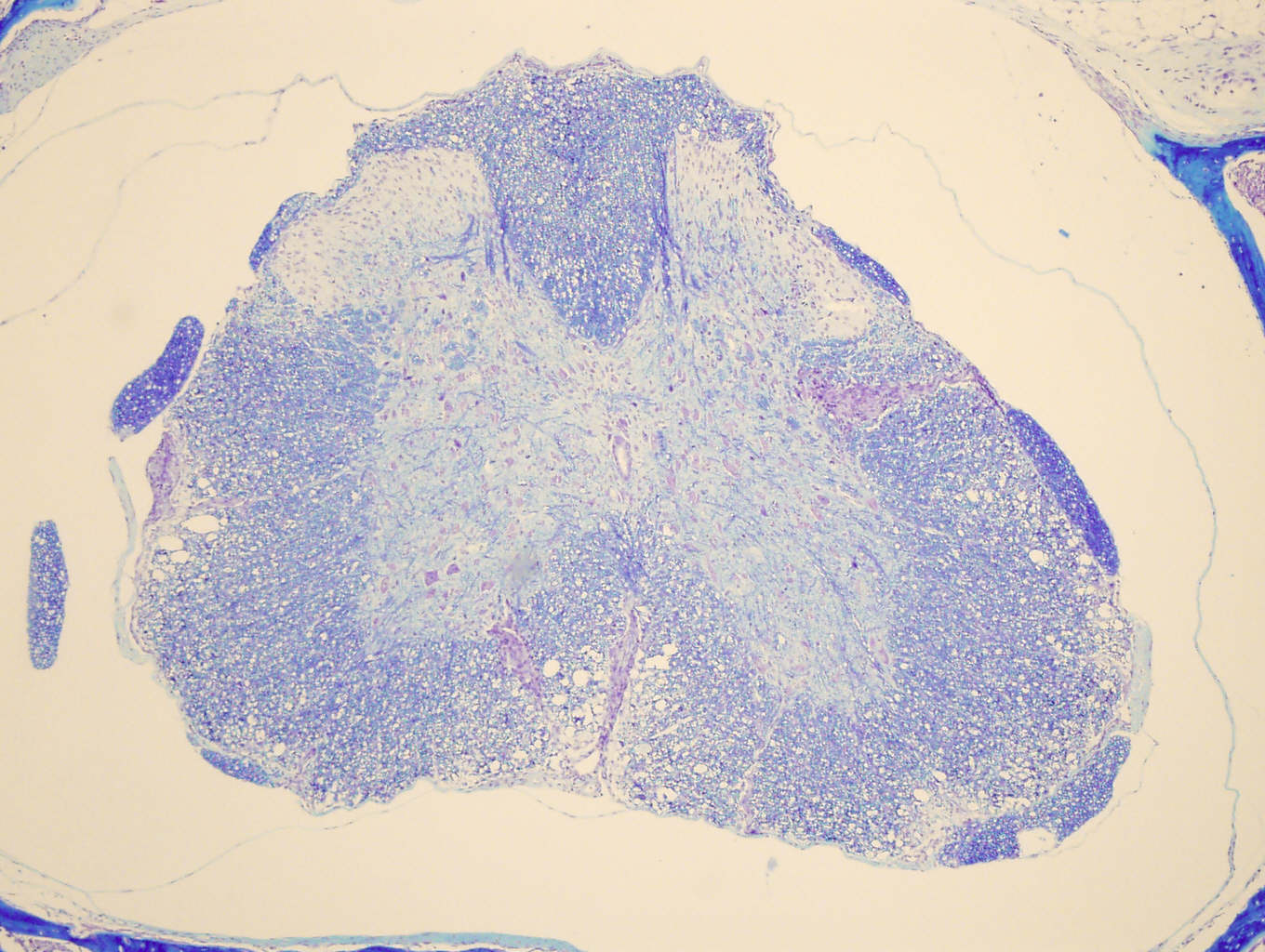
**

**Figure S11. H&E stained transverse section of lumbar spinal cord of mouse 1-1 (100x).**

Multiple inflammatory foci and multifocal inflammatory lesions are present in white matter, and parenchyma of the white matter (examples indicated by arrows) forming what appears like a ring-shaped inflammatory lesion. There is also some vacuolation in the white matter that is consistent with edema and demyelination.

**
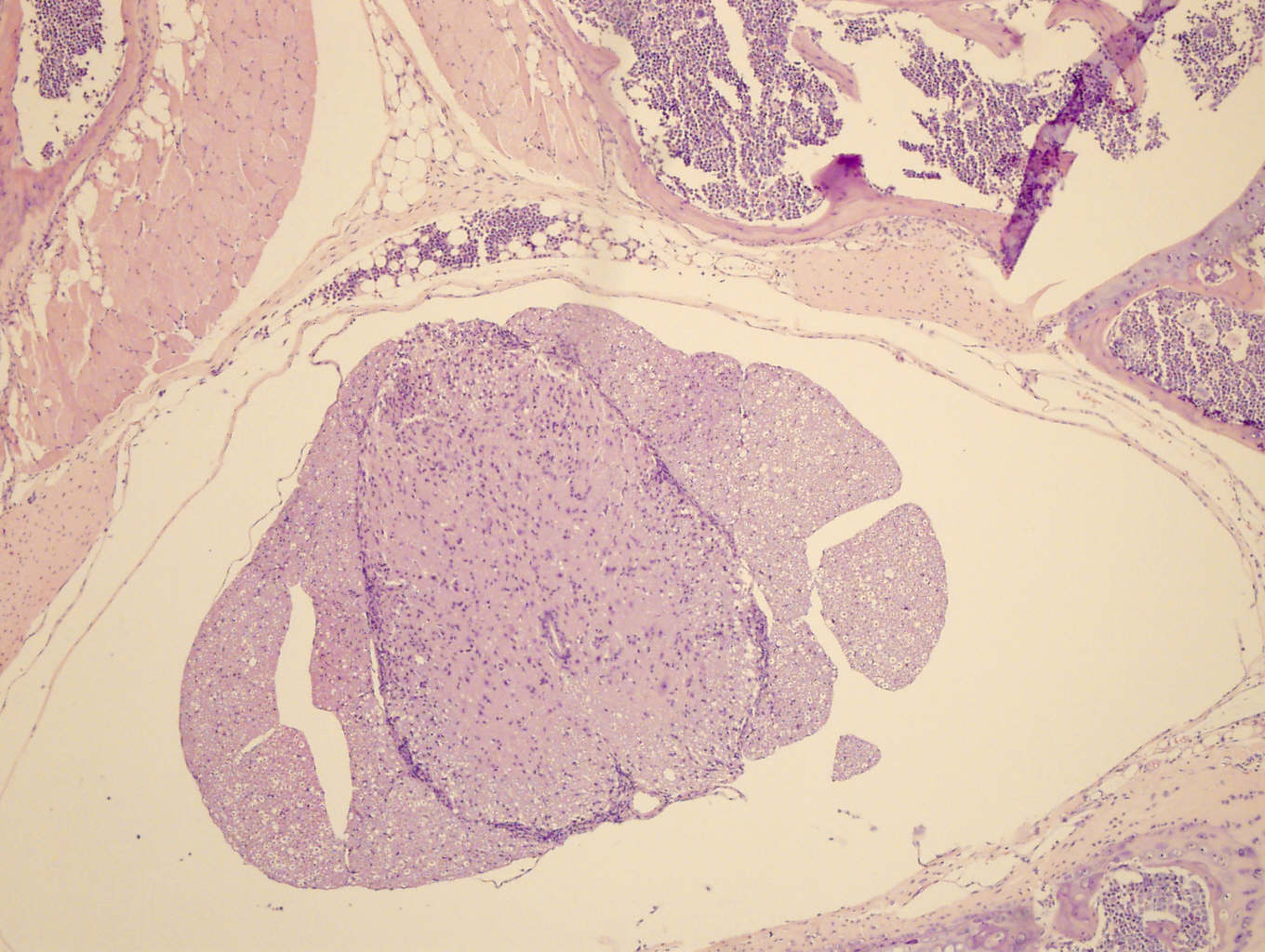
**

**Figure S12. Luxol fast blue stained section of lumbar spinal cord, mouse #1-1 (100x).**

This section is adjacent to the H&E stained section shown above. Areas of demyelination are visible within white matter (lighter blue stained areas, in the outer parts of the spinal cord as examples in boxed areas).

**
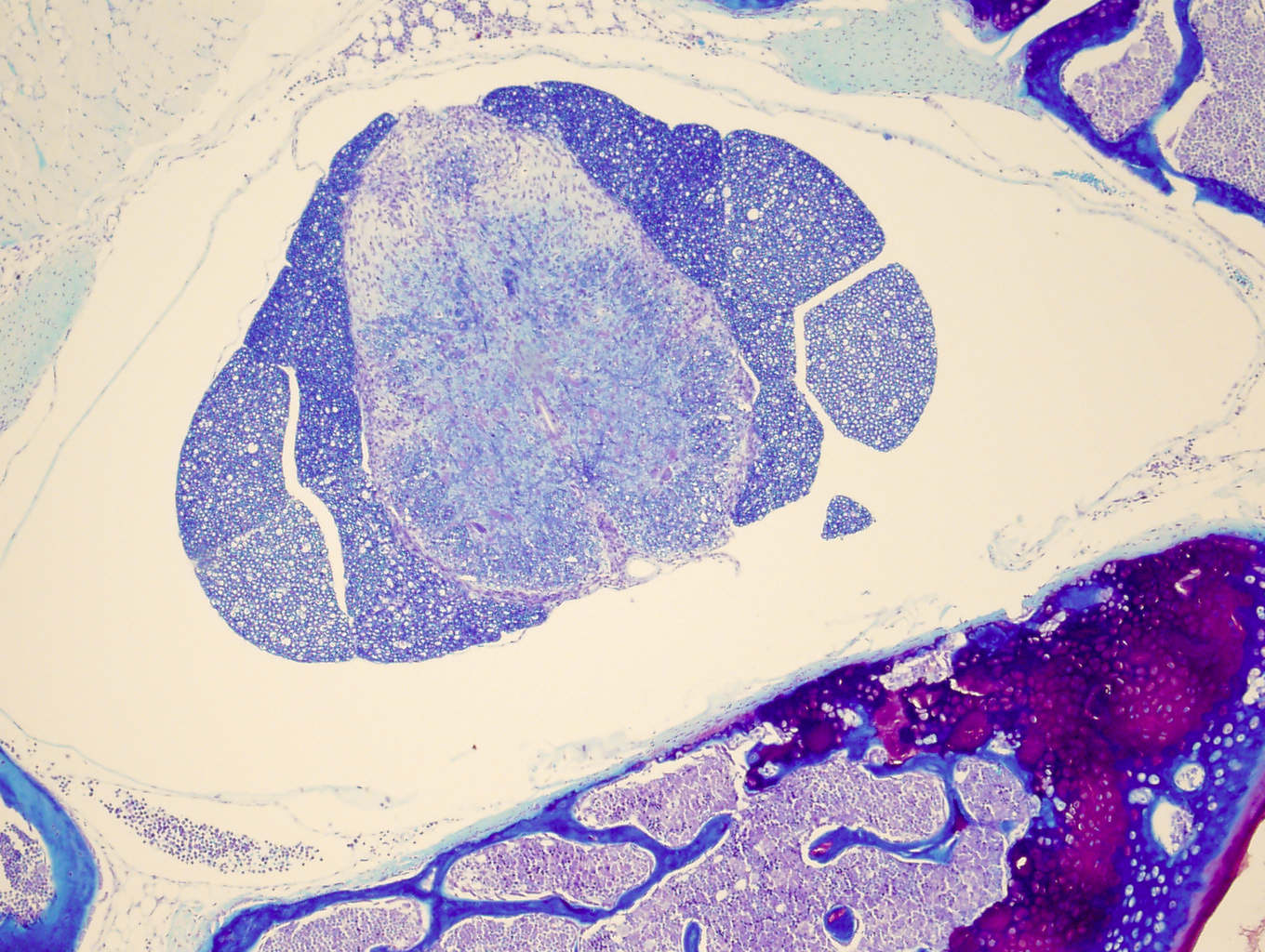
**

**Figure S13. H&E stained transverse section of cervical spinal cord of mouse 1-2 (100x).**

Multiple inflammatory foci and some multifocal inflammatory lesions are present in the leptomeninges, around blood vessels in the leptomeninges and white matter, and parenchyma of the white matter (as indicated by arrows). There also is vacuolation in the white matter that is consistent with edema and demyelination (example in boxed area).

**
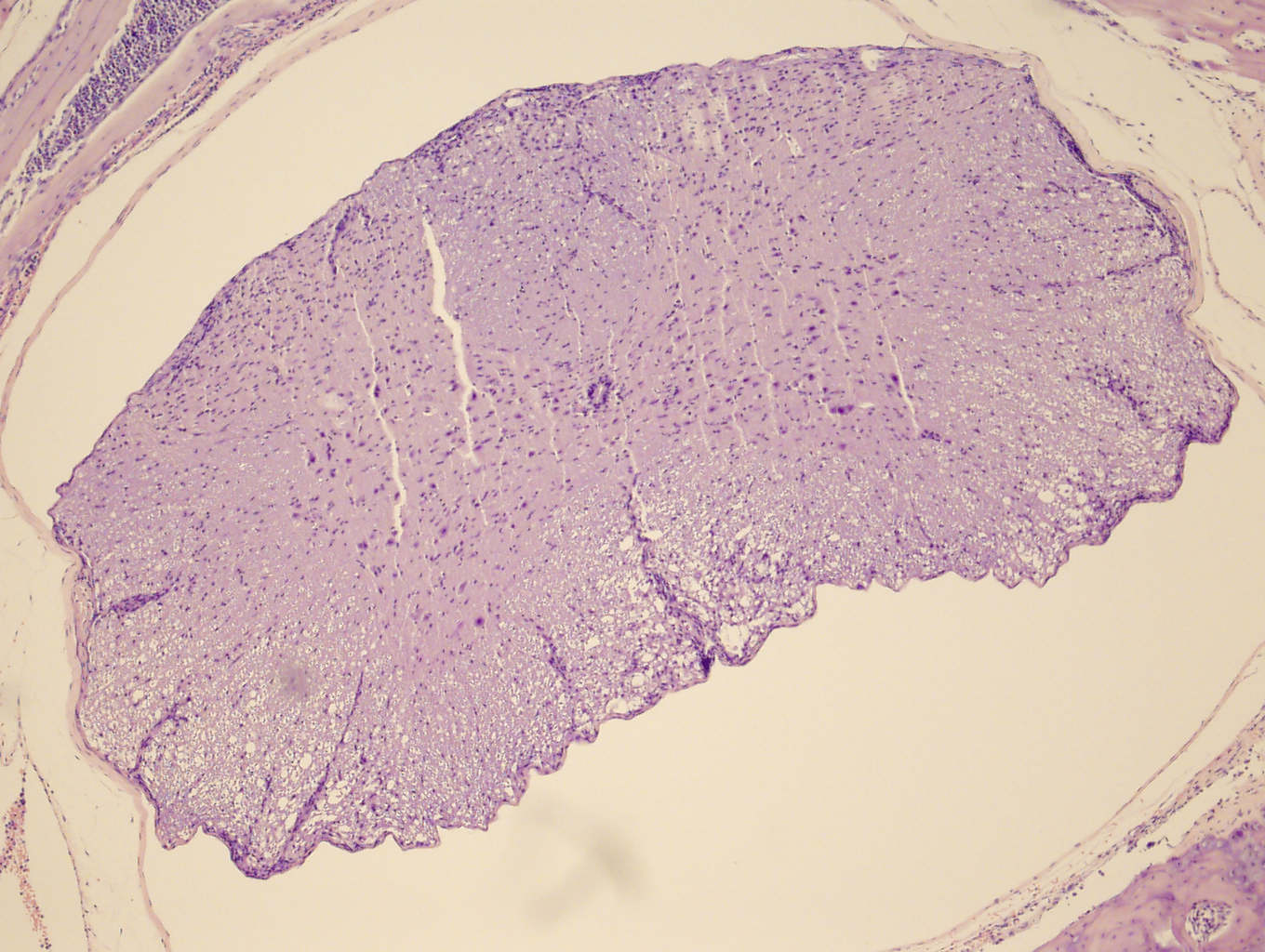
**

**Figure S14. Luxol fast blue stained section of cervical spinal cord, mouse #1-2 (100x).**

This section is adjacent to the H&E stained section shown above. Areas of demyelination are visible within white matter (lighter blue stained areas, in the outer parts of the spinal cord as indicated by arrows).

**
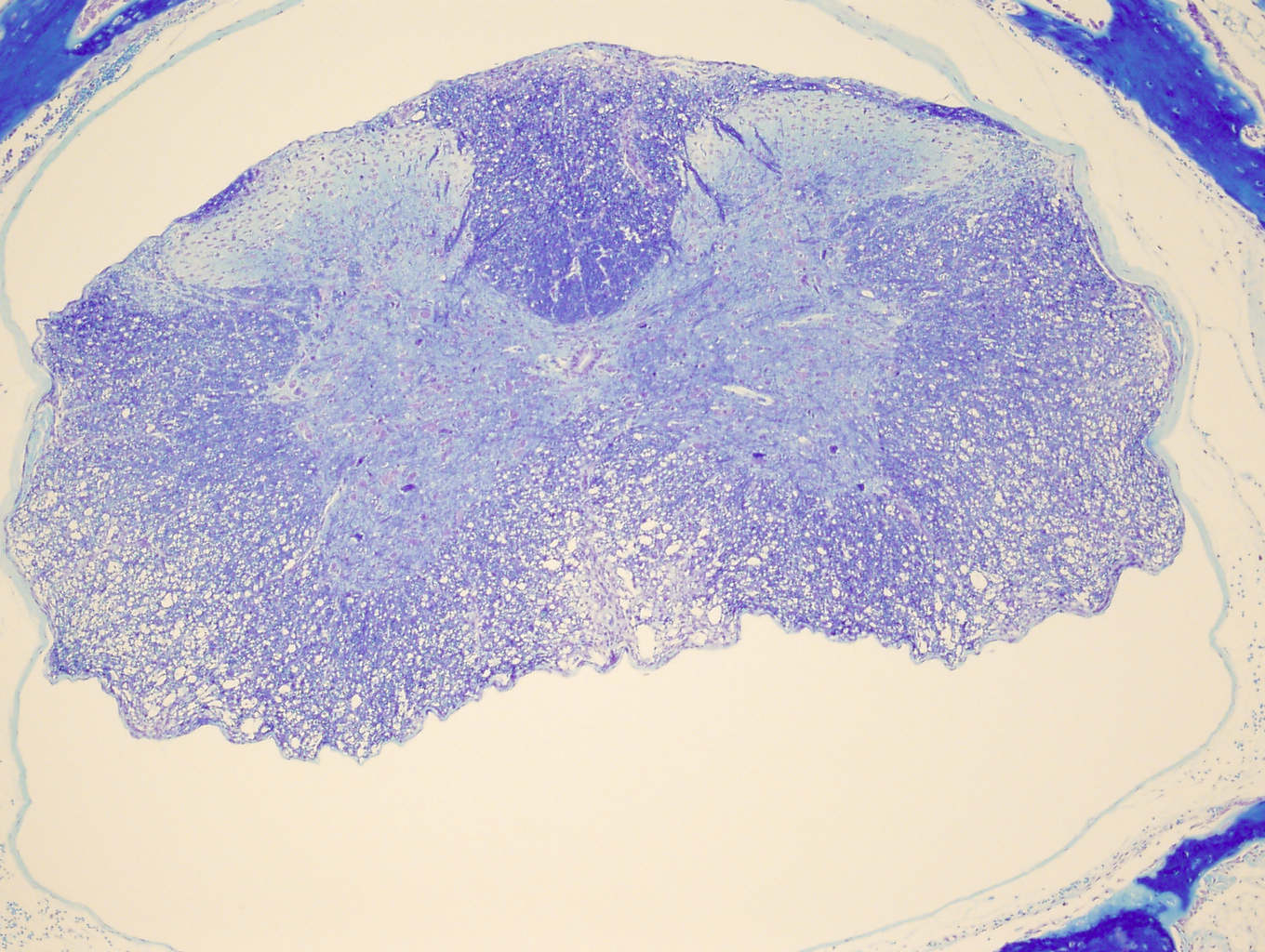
**

**Figure S15. H&E stained transverse section of thoracic spinal cord of mouse 1-2 (100x).**

Multiple inflammatory foci are present in the leptomeninges, around blood vessels in the leptomeninges and white matter (example in boxed area). There also is vacuolation in the white matter that is consistent with edema and demyelination (example of area within ellipse).

**
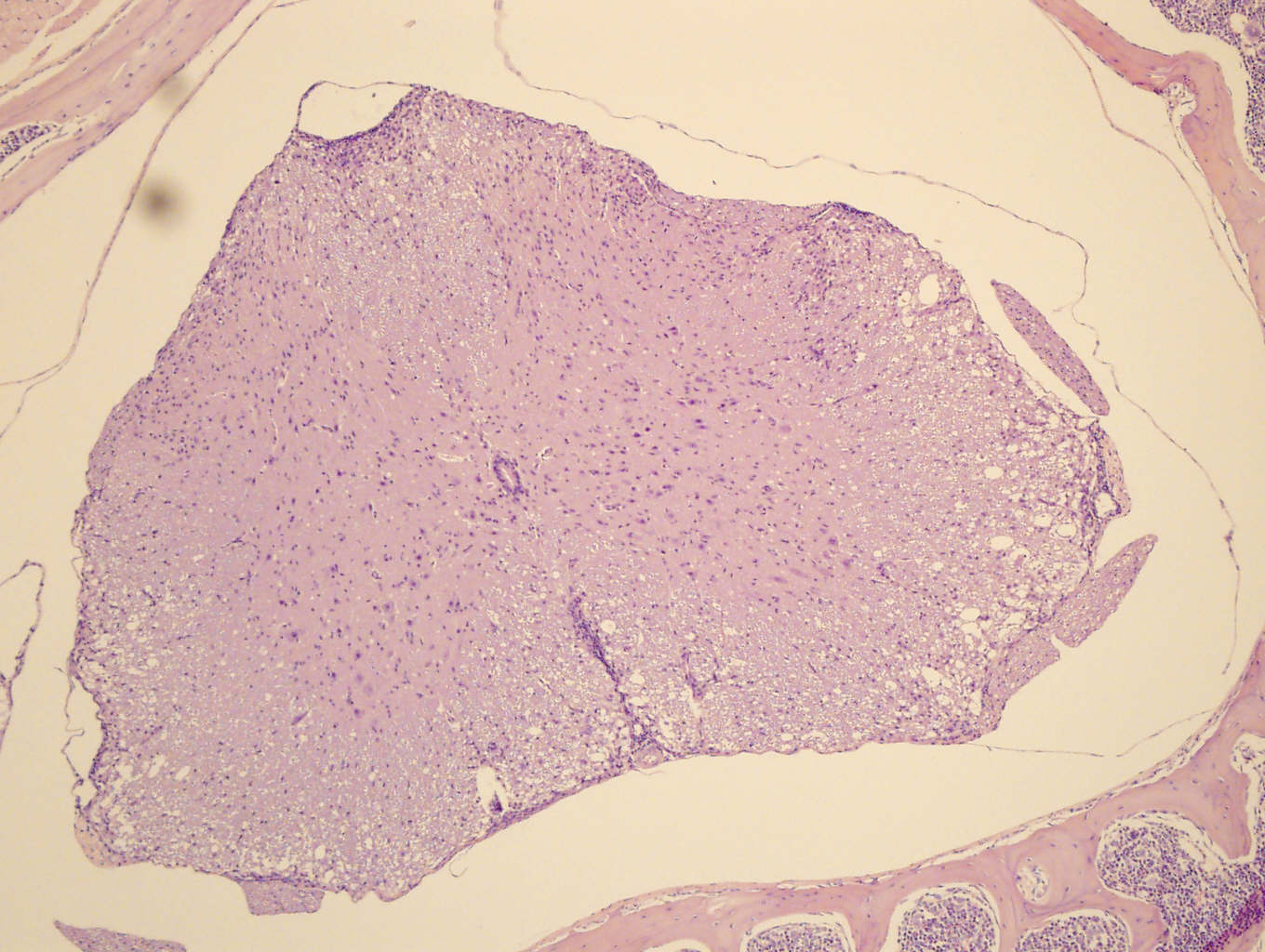
**

**Figure S16. H&E stained section of thoracic spinal cord, mouse #1-2 (400x).**Detail of the above slide (Boxed area). An inflammatory lesion is shown**.**

**
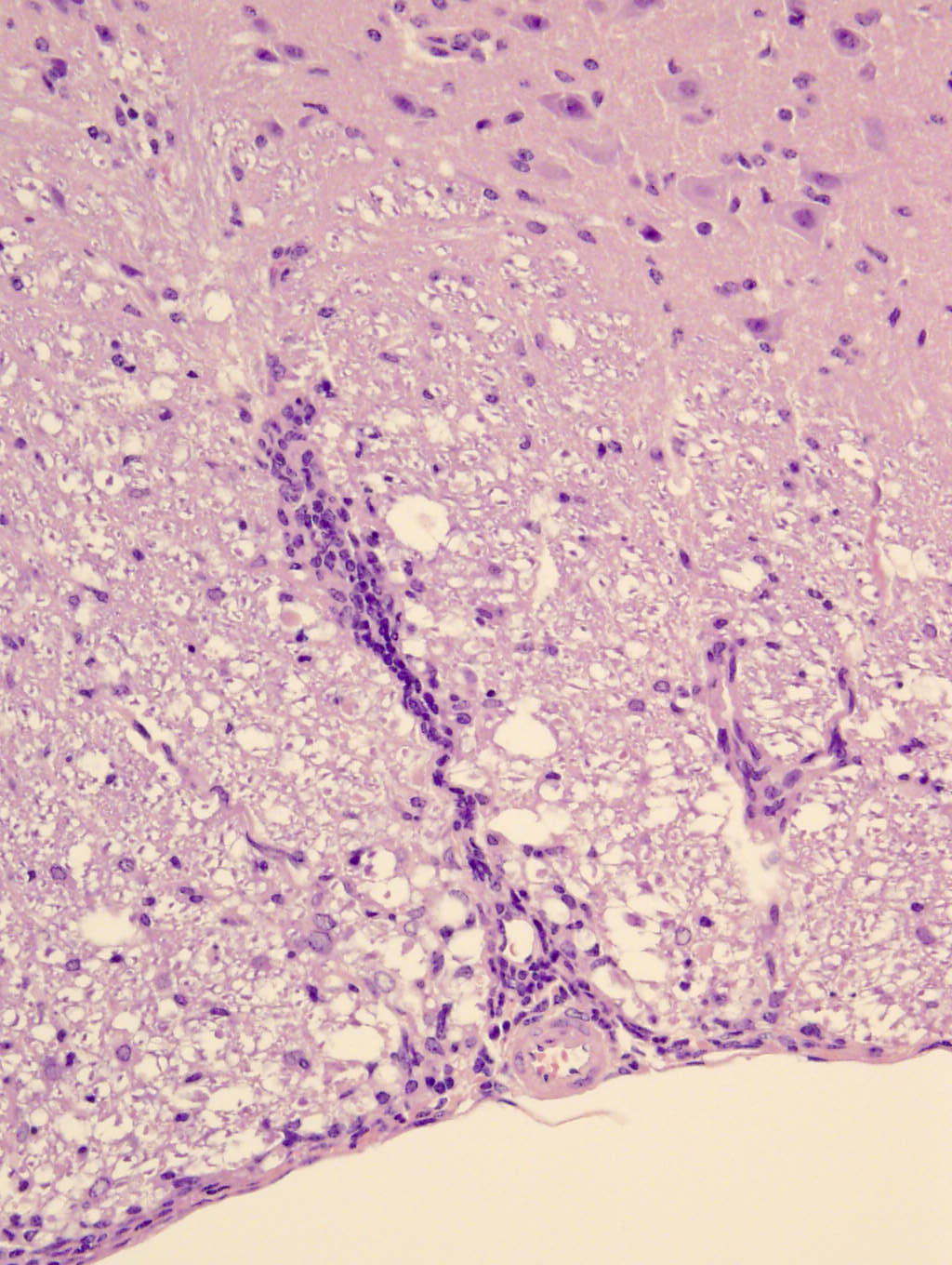
**

**Figure S17. Luxol fast blue stained section of thoracic spinal cord, mouse #1-2 (100x).**

This section is adjacent to the H&E stained section shown above. Areas of demyelination are visible within white matter (lighter blue stained areas, in the outer parts of the spinal cord).

**
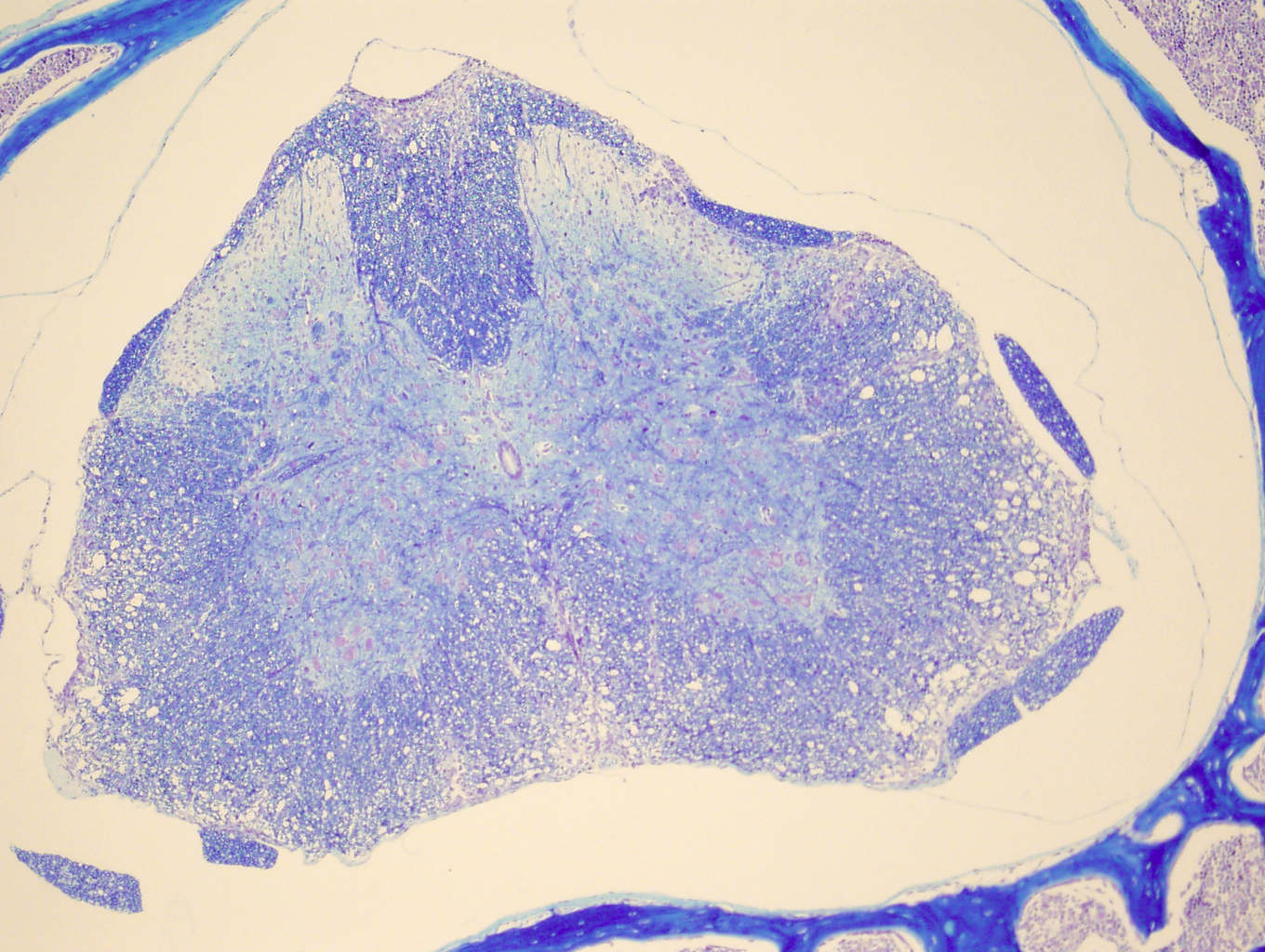
**

**Figure S18. H&E stained transverse section of lumbar spinal cord of mouse 1-2 (100x).**


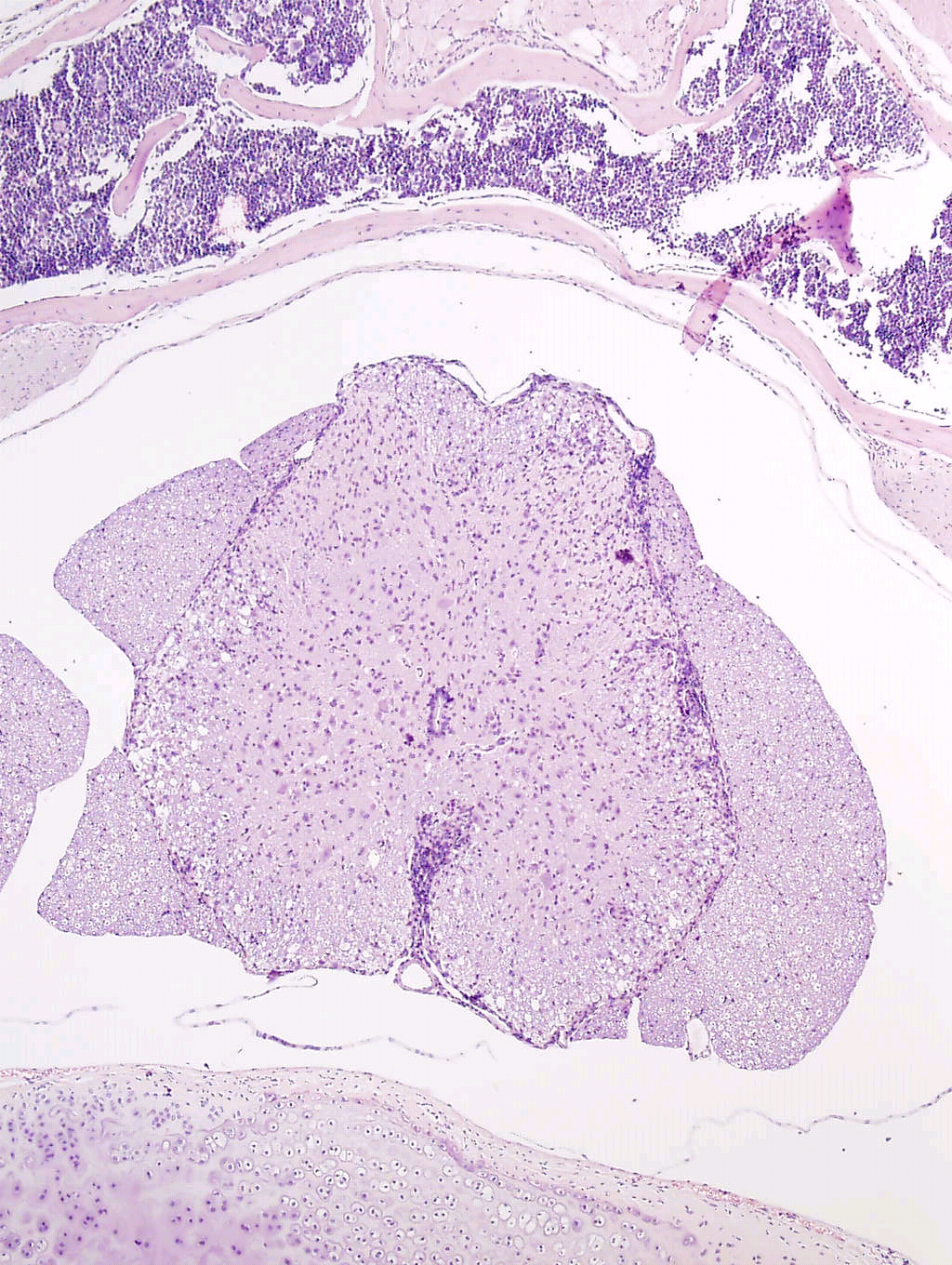
Multiple inflammatory foci and multifocal inflammatory lesions are present in white matter, and parenchyma of the white matter, forming what appears like a ring-shaped inflammatory lesion (examples in boxed areas). There is also some vacuolation in the white matter that is consistent with edema and demyelination.

**Figure S19. Luxol fast blue stained section of lumbar spinal cord, mouse #1-2 (100x).**

This section is from the same block of tissue as the H&E stained section shown above. Areas of demyelination are visible within white matter (lighter blue stained areas as boxed examples, in the outer parts of the spinal cord).

**
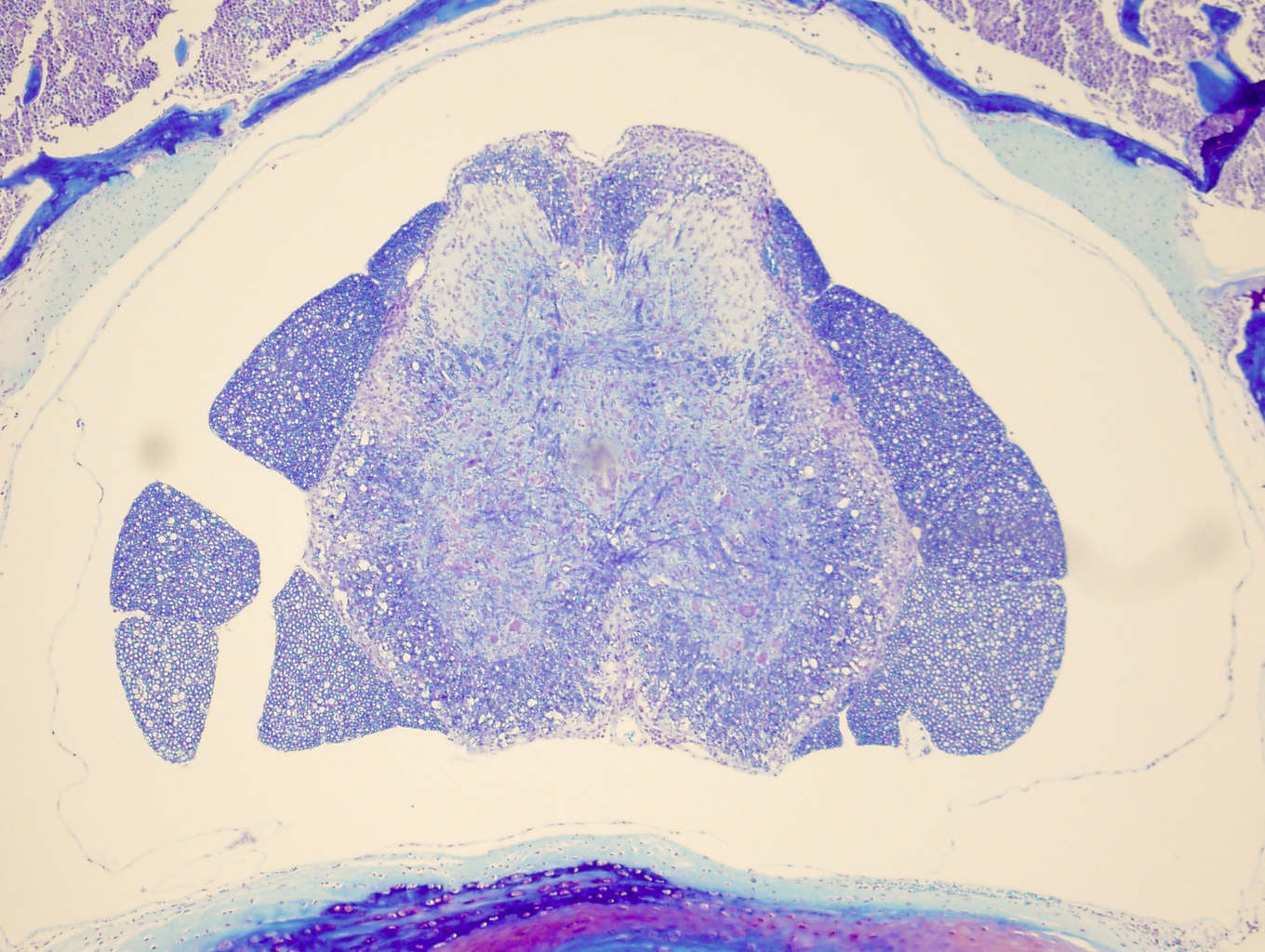
**

**Figure S20. H&E stained transverse section of lumbar spinal cord of mouse 2-5 (100x).**

One large inflammatory focus is present in the white matter (Box). There is some vacuolation the white matter around the inflammatory focus.

**
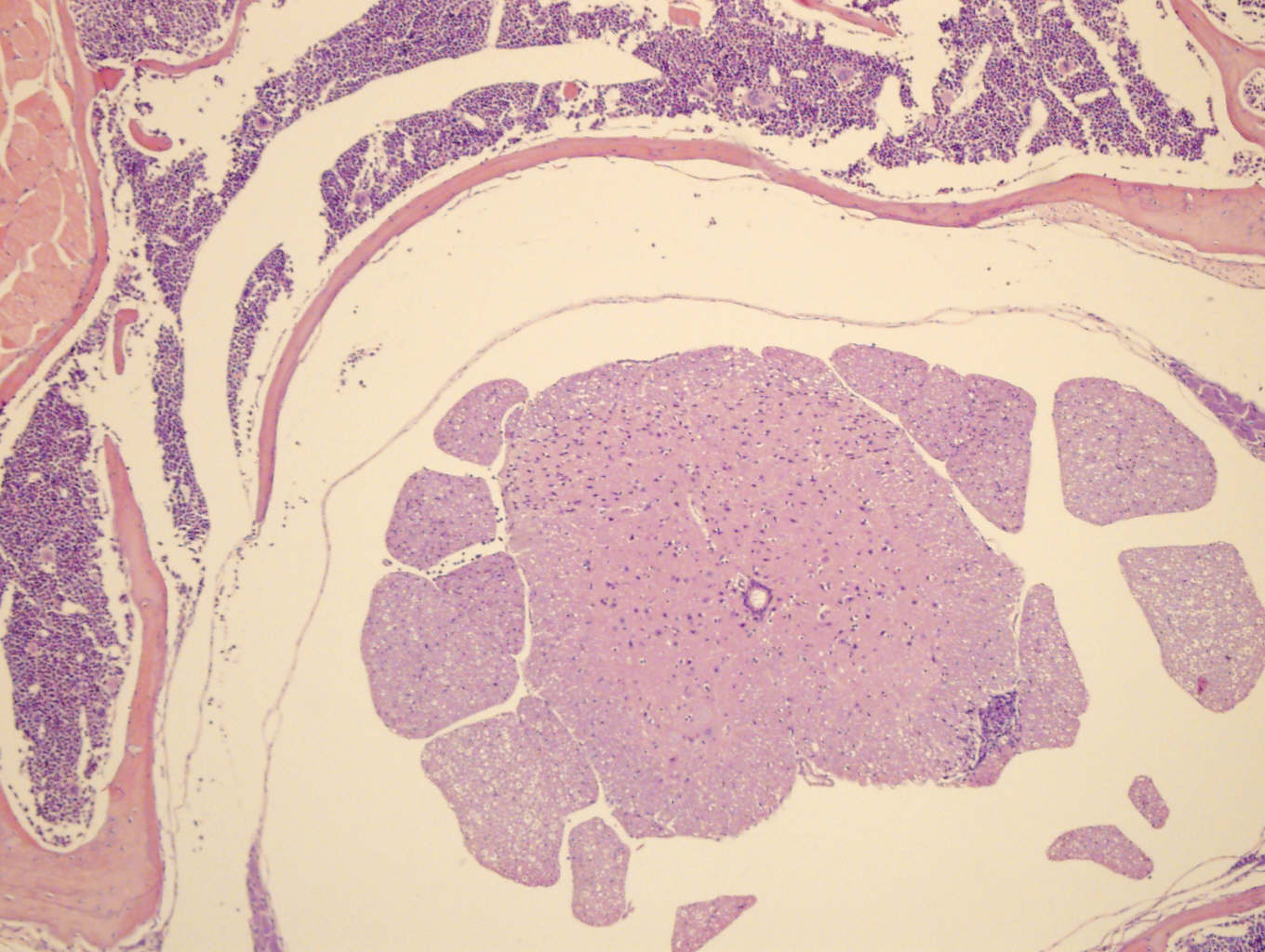
Figure S21. Luxol fast blue stained section of lumbar spinal cord, mouse #2-5 (magnification 100x).**

This section is adjacent to the H&E stained section shown above. There is no significant demyelination within white matter.

**
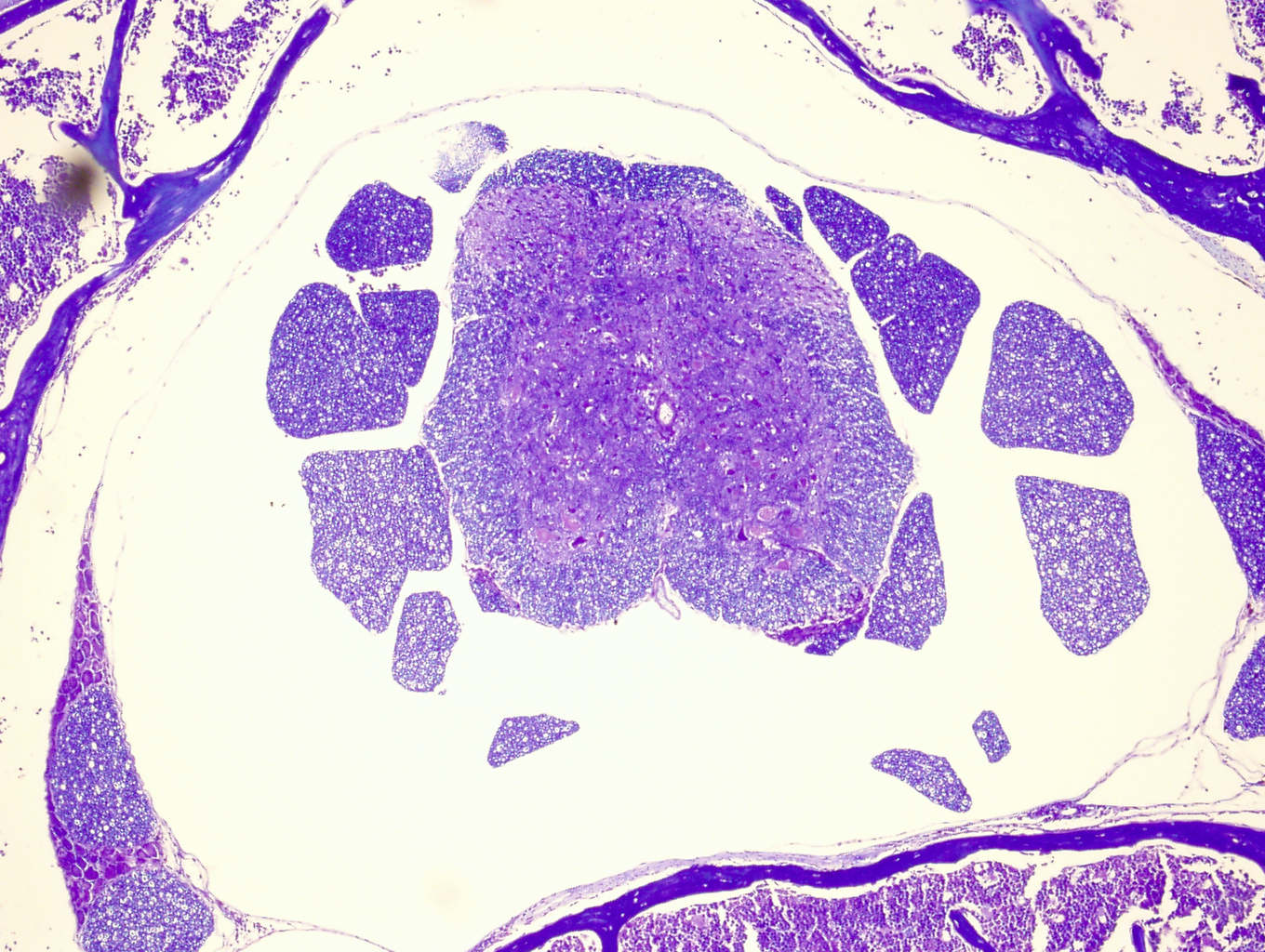
**

**II.3 Appendix (raw data).**

**Table S.A1. Body weights during the study.**

**Table S.A2. Body weights as % of body weight on Day -1.**

**Table S.A3. EAE scores during the study.**

**Table S.A3 (cont). EAE scores during the study (cont)**

**III. Recombinant envelope protein:**

**III.1: Origin and structure:**

Recombinant Env protein was expressed from plasmid pV14 encompassing the full-length *orf* of HERV-W env gene encoding the MSRV envelope (58 KDa, 542 aminoacids) cloned from MSRV virion RNA (Env; GenBank no. [AF331500.1](http://www.ncbi.nlm.nih.gov/entrez/viewer.fcgi?val=AF331500.1); Perron H et al. *Virology* 2001;287:321-332). Env-SU is a 33-kDa and 449 amino acids extracellular portion of the full-length MSRV envelope protein (542 amino acids).

Figure S22 : Structure of complete Env protein, corresponding domains and ENV-SU region expressed as recombinant protein.

**III.2. QC reports on production and purification of endofree recombinant MSRV-Env protein.**

**III.1.a Env-SU:**

**III.1.b Full Length MSRV-Env (Env-T):**

Imprint date: January, the 5th 2011

Construct reference:

Env_pv41-pQE80K/1

Protein batches reference: ENV T-101129

1. Protein sequence: 548 aa (His-tagged) MALPYHTFLFTVLLPPFALTAPPPCCCTTSSSPYQEFL WRTRLPGNIDAPSYRSLSKGNSTFTAHTHMPRNCYNSA TLCMHANTHYWTGKMINPSCPGGLGATVCWTYFTHTSM SDGGGIQGQAREKQVKEAISQLTRGHSTPSPYKGLVLS KLHETLRTHTRLVSLFNTTLTRLHEVSAQNPTNCWMCL PLHFRPYISIPVPEQWNNFSTEINTTSVLVGPLVSNLE ITHTSNLTCVKFSNTIDTTSSQCIRWVTPPTRIVCLPS GIFFVCGTSAYHCLNGSSESMCFLSFLVPPMTIYTEQD LYNHVVPKPHNKRVPILPFVIRAGVLGRLGTGIGSITT STQFYYKLSQEINGDMEQVTDSLVTLQDQLNSLAAVVL QNRRALDLLTAKRGGTCLFLGEERCYYVNQSRIVTEKV KEIRDRIQCRAEELQNTERWGLLSQWMPWTLPFLGPLA AIIFLLLFGPCIFNFLVKFVSSRIEAVKLQIVLQMEPQ MQSMTKIYRGPLDRPARLCSDVNDIEVTPPEEISTAQP LLHSNSVGSSHHHHHH
2. Properties:

Abs 0.1% (1g/L): 1.21

Molecular weight: 61.44 kDa Theoretical pI: 8.6

Number of Met: 13 Number of Cys: 20

1. Production method:

- Strain: BL21 E. coli strain
- Medium: Riesenberg medium with 30µg/mL kanamycin

- Culture conditions: 4L of fermentation were performed at 37°C, with glucose feed

until the optical density reached 90. Induction was done with 1mM IPTG during 4H. Cells were then harvested after 12

hours at 20°C (final OD =95).

- Cell harvest: centrifugation at 5000xg, 15 minutes, 4°C.
- Intermediate storage: Cell pellets were

stored at -80°C until extraction (approx. 6 months)

1. Extraction method:
   - Pellet corresponding to 1L of fermentation culture was resuspended in 1050 mL of lysis buffer (20mM Tris- HCl pH 7.5, 150mM NaCl; 1µg/mL leupeptin; 1µg/mL pepstatin; 2mM MgCl2; lysozyme 100µg/mL) and incubated for 1 hour at 8°C under shaking.
   - After 3 cycles of cell disruption, 5900 UI of Benzonase Nuclease was added

(Novagen) and lysate was incubated for 1 hour at RT under shaking.

- - Soluble and insoluble proteins were separated by centrifugation at 46000g,

for 30min at 4°C.

- - Pellets solubilization was done in 305mL of 20mM Tris-HCl pH 7.5, 150mM NaCl, 2M urea, 1.5% SDS, 50mM β-

mercaptoethanol, 1µg/mL leupeptin; 1µg/mL pepstatin under shaking overnight.

- - Solubilized proteins were separated by centrifugation at 46000g, for 30 min at 10°C.
  - Solubilized proteins were diluted 5 times

in buffer 20mM Tris-HCl pH 7.5, 150mM NaCl, 1.5% SDS in order to decrease β- mercaptoethanol concentration.

1. Purification method: Affinity chromatography:

Purification was performed onto affinity resin

Ni-Sepharose 6 Fast Flow (CV=40mL, Amersham GE Healthcare).

- - Equilibration buffer: 20mM Tris-HCl pH

7.5, 150mM NaCl, 500mM urea, 1.5%

SDS, 10mM β-mercaptoethanol.

- - Load: 1450mL of solubilized proteins.
  - Wash1: 15 CV of equilibration buffer.
  - Wash2: 15 CV of equilibration buffer at 5mM imidazole
  - Elution steps: 10 CV of equilibration

buffer at 50mM imidazole and 5 CV of equilibration buffer at 150mM imidazole.

- - Pool of the 50mM imidazole elution

fraction (total volume: ~75mL).


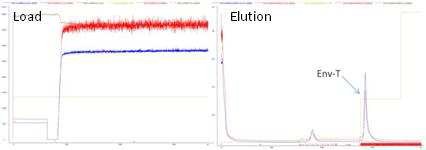


Ni Sepharose chromatogram.

1. Dialysis

75mL were dialyzed in three baths of 5L of 20mM Tris-HCl pH 7.5, 150mM NaCl, 1.5%

SDS, 10mM DTT, with a cut-off of 12-14

MWCO (2 x 4h, 1 x 12h).

1. Batches EnvT characteristics:

Batch ENV T-101109

Buffer: Tris-HCl 20 mM pH 7.5, NaCl 150mM, SDS 1.5%, DTT 10mM

Volume: 37 x 2mL

Concentration (evaluated by densitometry on Coomassie blue stained SDS-PAGE against BSA): approx 0.5mg/mL

Quantity: 37mg

**Endotoxin level: <5 EU/mL**

Purity ~84%

N-ter sequencing: A L P Y H T F L F T in accordance with the sequence of the N- terminal end of EnvT from the second residue Storage: -80°C


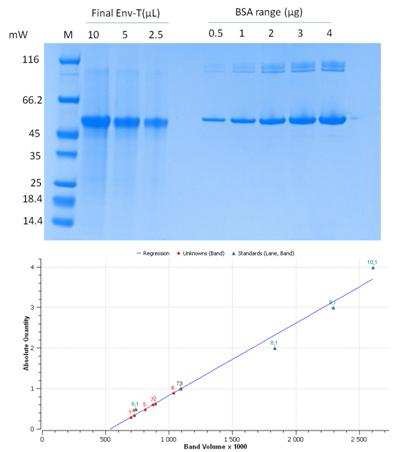


Final product analysis by densitometry on Coomassie blue stained SDS-PAGE, using Image Lab software (Bio-Rad); Sample concentration is evaluated by a linear regression of calibration range tracks (concentration: 0.5 mg/mL).


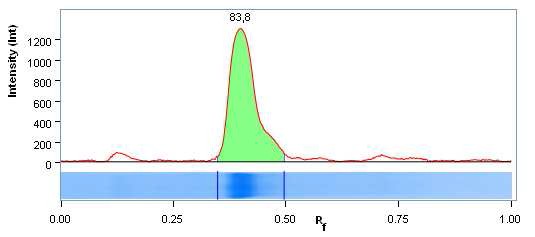


Final product analysis by densitometry on Coomassie blue SDS-PAGE using Image Lab software (Bio- Rad), showing purity of Env-T (% of whole lane density; purity: at least~84%)


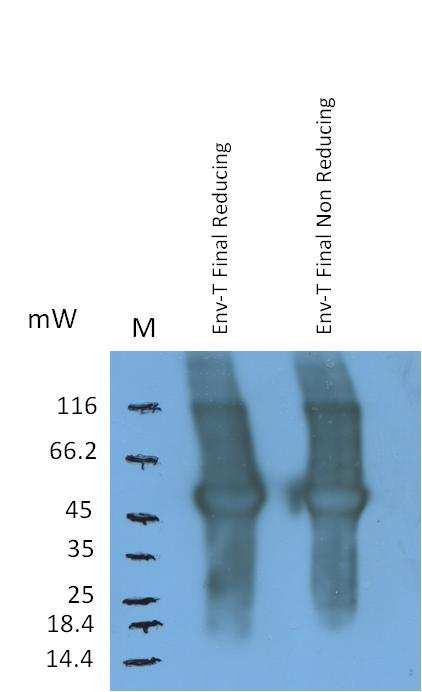


4-12% SDS-PAGE, reducing and non-reducing conditions, followed by Western blot using 21G8 antibody.

1. Storage:

Unopened vials should be stored at -80°C.
